# Efficient characterization of high-dimensional parameter spaces for systems biology

**DOI:** 10.1186/1752-0509-5-142

**Published:** 2011-09-15

**Authors:** Elías Zamora-Sillero, Marc Hafner, Ariane Ibig, Joerg Stelling, Andreas Wagner

**Affiliations:** 1 Department of Biochemistry, University of Zurich, Zurich, Switzerland; 2 Department of Biosystems Science and Engineering, ETH Zurich, Zurich, Switzerland; 3 Swiss Institute of Bioinformatics, Lausanne, Switzerland; 4 School of Computer and Communication, EPFL, Lausanne, Switzerland; 5 The Santa Fe Institute, Santa Fe, New Mexico, USA; 6 Department of Biology, University of New Mexico, Albuquerque, New Mexico, USA

## Abstract

**Background:**

A biological system's robustness to mutations and its evolution are influenced by the structure of its viable space, the region of its space of biochemical parameters where it can exert its function. In systems with a large number of biochemical parameters, viable regions with potentially complex geometries fill a tiny fraction of the whole parameter space. This hampers explorations of the viable space based on "brute force" or Gaussian sampling.

**Results:**

We here propose a novel algorithm to characterize viable spaces efficiently. The algorithm combines global and local explorations of a parameter space. The global exploration involves an out-of-equilibrium adaptive Metropolis Monte Carlo method aimed at identifying poorly connected viable regions. The local exploration then samples these regions in detail by a method we call multiple ellipsoid-based sampling. Our algorithm explores efficiently nonconvex and poorly connected viable regions of different test-problems. Most importantly, its computational effort scales linearly with the number of dimensions, in contrast to "brute force" sampling that shows an exponential dependence on the number of dimensions. We also apply this algorithm to a simplified model of a biochemical oscillator with positive and negative feedback loops. A detailed characterization of the model's viable space captures well known structural properties of circadian oscillators. Concretely, we find that model topologies with an essential negative feedback loop and a nonessential positive feedback loop provide the most robust fixed period oscillations. Moreover, the connectedness of the model's viable space suggests that biochemical oscillators with varying topologies can evolve from one another.

**Conclusions:**

Our algorithm permits an efficient analysis of high-dimensional, nonconvex, and poorly connected viable spaces characteristic of complex biological circuitry. It allows a systematic use of robustness as a tool for model discrimination.

## Background

High-throughput experimental technologies have allowed biology to generate huge amounts of data. The enormity of these data sets permits a systemic view of the cell [1]. In this new framework mathematical models are immensely useful as compact representations of data [2], and as highly structured hypotheses that include underlying mechanisms of the processes under study. These models often consist of large systems of ordinary differential equations that govern the kinetics of proteins, mRNAs, and small molecules. 

Mathematical modeling in biology faces several challenges that arise from uncertainty about relevant parameters. For example, the chemical reactions and the corresponding kinetic equations governing any one biological system are only partially known [3,4]. Also, finding accurate numerical values for model parameters is virtually impossible, because many biochemical parameters cannot be measured directly. In addition, evolutionary processes can cause parameters to vary on evolutionary time scales, yet preserve system function. Thus, even a perfect mathematical model of an individual system might have limitations in describing other individuals of the same population that are sufficiently diverse genetically or epigenetically. In sum, it is often of limited use to identify a single best set of parameters for any one biochemical system. However, one can focus on a viable parameter *space *instead. This viable space is a subset of a space of biochemical parameters, where a model maintains a desirable behavior. Values of these parameters must lie inside the boundaries of this viable space for every organism in a population.

The investigation of viable spaces is closely linked to the analysis of robustness in biology. We here define robustness as the persistence, under perturbations, of a behavior that is characteristic for a system [5]. When focusing on robustness to changes in biochemical parameters that define system behavior, a biological system's robustness is a reflection of the topology and size of its viable space [6,7]. The volume of the viable space indicates the "amount" of parameter combinations that allow a system's desired behaviour. A small viable volume forces a precise tuning of biochemical parameters. in contrast, a large viable volume allows a system to successfully face changes in environmental conditions, because its parameters can change, sometimes by orders of magnitude, without impairing its function. Hence, robustness is associated with larger viable volumes.

The geometry of viable spaces also plays an important role in a system's robustness. Geometries that permit moderate parameter fluctuations without leaving the viable volume enhance robustness. In evolutionary terms, different ways of performing the same function - for instance, by conserved pathways with homologous yet different proteins [8] - can be traced back to a common ancestor and are thus "reachable" from each other [9]. A connected viable volume improves a system's evolvability and allows neutral evolutionary trajectories that may drive the system towards viable parameter points with high local robustness. Therefore, the robustness of a biological system can be a reflection of the geometry and size of its viable space.

A final motivation to characterize viable spaces comes from model building itself. As we pointed out above, some relevant components and interactions in cellular networks are typically unknown. It follows that the structure of mathematical models describing these networks contains uncertainties. These uncertainties may lead to qualitatively different models that match experimental observations equally well. In this case, robustness can be used as a tool to discriminate between more and less plausible models. Everything else being equal, a model can be considered superior if it is more robust than other plausible models [5,8]. 

The use of robustness for model discrimination raises the problem of how to measure robustness. Most robustness analyses in the literature are local (e.g. see [10-12] and references therein). They use a specific set of parameters, and their results do not reflect model behavior under all possible viable parameter sets. Some nonlocal approaches alter one or two parameters, and use bifurcation analysis to characterize the regions of a parameter space with similar qualitative model behavior [8,13-18]. These methods have serious limitations whenever multiple parameters have unknown values, which is usually the case. To address these limitations, a third group of techniques [7,19] use "glocal" approaches [20]. In a first "global" step of their analysis, these techniques obtain a sample of parameters from the viable space, and then, in a "local" analysis, they study the local robustness around every element of this set. In this way, they compute nonlocal measures of robustness, but they also face the problem of acquiring a large and statistically representative sample of viable parameter points. Therefore, they need efficient global methods to sample the viable space.

The main challenges for global methods typically result from the fact that parameter spaces can have many dimensions and a complex geometry, about which one has little prior knowledge. To characterize a viable space, some authors perform uniform sampling of the whole parameter space to identify regions where a model displays the desired behavior [8,21-25]. Determining this behavior typically involves integration of the model equations, which can become computationally very expensive when done for large samples. Even more fundamentally, the "curse of dimensionality" [26] makes the fraction of the whole parameter space occupied by viable parameters decrease exponentially with increasing dimension, i.e., increasing number of parameters. Therefore, "brute force" uniform sampling becomes quickly infeasible as model complexity increases. To avoid this problem, Hafner *et al. *[20] developed an algorithm that explores a parameter space by iterative Gaussian sampling. Briefly, in every iteration, this method determines the mean value and the covariance matrix of the identified viable points in parameter space to guide further sampling. However, the algorithm is only efficient when the viable region is convex and when enough viable points are found in each iteration.

Here, we propose an algorithm that overcomes these limitations. Specifically, it can efficiently characterize nonconvex and poorly connected viable spaces. The algorithm consists of two steps, namely a coarse-grained sampling of the viable space, which in turn delivers starting points for a finer-grained exploration. The sampled points also define a domain for subsequent volume computations by Monte Carlo integration, and for acquisition of a large set of uniformly distributed viable points. After describing the algorithm, we analyse a synthetic test problem involving a nonconvex and poorly connected viable space. This analysis will show that in high dimensional spaces our algorithm converges faster and identifies a larger proportion of the viable space than uniform sampling and Hafner's method. Moreover, in contrast to uniform sampling and Hafner's algorithm, whose performances scale exponentially with the number of dimensions, our algorithm's performance scales linearly with the number of dimensions. Subsequently, we illustrate an application of our method to a biochemical circuit. To this end, we focus on a simplified model of biochemical oscillators with positive and negative feedback loops [27,28], in order to investigate the contributions of individual control loops to the robustness of oscillations in a narrow range of frequencies. Our algorithm allows us to characterize the nonconvex viable space of this model. In spite of the model's simplicity, the geometry of this space shows well known properties of circadian oscillators. Specifically, it indicates that model topologies with an essential negative feedback loop and a nonessential positive feedback loop provide the most robust fixed period oscillations, as has been observed in different models of circadian oscillators [19,29-32]. In addition, the connectedness of the model's viable space suggests that biochemical oscillators with varying topologies can evolve from one another.

## Methods

### Viable regions

**Figure 1:**
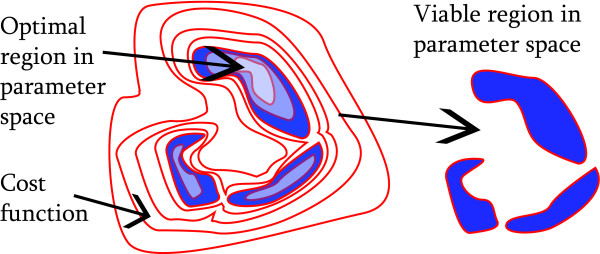
**Hypothetical cost function and viability condition**. Contour plot (red curves) of a generic cost function in a 2-dimensional parameter space. Blue areas correspond to the viable space defined by a threshold on the cost function. Some regions in the viable space may have different cost, indicated by different shades of blue in the left panel.

Given a model that involves *d *parameters, we define a parameter space as

(1) $$\Theta^{d}=\Theta_{1}\times \Theta_{2}\times \cdot \cdot \cdot \times \Theta_{d},$$

where Θ*_i _*is the interval of the real numbers ℝ for which the parameter *θ_i _*is defined. We call the *d*-tuple *θ *= (*θ*_1_, *θ*_2_, ..., *θ_d_*) ∈ Θ*^d ^*a parameter point. It represents a configuration of the biochemical parameters involved in the model (Figure 1). In addition, each parameter point has an associated value of a cost function

(2) $$E\left(\theta \right):\Theta^{d}\to {\mathbb{R}}^{+},$$

that reflects how well a model produces a behavior under consideration. For a given *θ*, the lower the value of *E*(*θ*) the better the model behaves.

A parameter point *θ *is viable if it fulfills the condition

(3) $$E\left(\theta \right)<E_{0},\;E_{0}>0,$$

that is, if the cost function does not exceed some positive threshold *E*_0_. For example, *θ *may imply a system behavior that allows an organism to survive or reproduce. The subset of parameter points *θ *∈ Θ*^d ^*for which (3) holds comprises the viable space [2,20].

### Out-of-equilibrium adaptive Monte Carlo sampling

**Figure 2:**
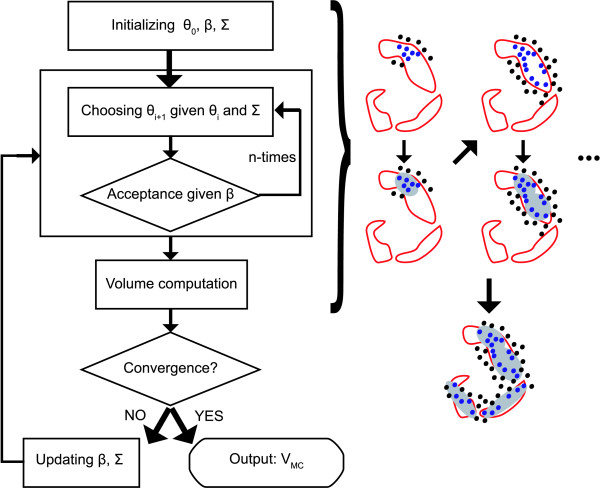
**Flowchart representing the basic scheme of the out-of-equilibrium adaptive Monte Carlo (OEAMC) algorithm**. Given an initial parameter point *θ*_0_, covariance matrix ∑ and *β*, the algorithm carries out *n *iterations in which every new parameter point is sampled from a normal distribution (4), and accepted or rejected based on Metropolis acceptances ratios (5). Every *n *iterations the viable points (blue and black points in the figure correspond to viable and nonviable sampled parameter points, respectively) found so far are grouped into clusters and the volume (grey areas in the figure) of ellipsoids that enclose the viable parameter points in each cluster is calculated. If the sum of these volumes converges the algorithm stops; if not, the covariance matrix ∑ and *β *are updated (6), and *n *new iterations are performed. The output of the algorithm is the set *V_MC _*which includes all the viable parameter points found.

We next describe our coarse-grained, global exploration of the viable space via an out-of-equilibrium adaptive Metropolis Monte Carlo sampling (OEAMC) (Figure 2).

The Metropolis algorithm was initially introduced to analyse thermodynamic systems [33]. However, it can also be applied to systems like those we study here. To do so, one must identify the parameter space Θ*^d ^*and the cost function *E*(*θ*) with a state space and with the energy of a thermodynamic system, respectively [34]. Moreover a parameter *β *has to be introduced in order to mimic the inverse of the temperature. This parallelism has been widely used in simulated annealing [35] and Metropolis Monte Carlo sampling [36-41].

This analogy allows us to use an adaptive selection probability with covariance matrix ∑

(4) $$g\left(\theta_{i}\to \theta \right)=\frac{1}{\sqrt{{\left(2\pi \right)}^{d}\left|\Sigma \right|}}\exp\left[-\frac{1}{2}\left(\theta -\theta_{i}\right)\Sigma^{-1}{\left(\theta -\theta_{i}\right)}^{\prime }\right],$$

in order to propose the transitions between parameter points, and Metropolis adaptive acceptance ratios

(5) $$A\left(\theta_{i}\to \theta \right)=\left\{\begin{array}{cc} \exp\left[-\beta \left(E\left(\theta \right)-E\left(\theta_{i}\right)\right)\right], & \text{if}\;E\left(\theta \right)-E\left(\theta_{i}\right)>0,\\ 1, & \text{otherwise,} \end{array}\right)$$

to accept or not those transitions.

Given *β *and ∑, the exploration starts from a known viable parameter point *θ*_0_. Then, from the current *θ*_0 _a new *θ *is constructed by sampling the distribution (4) centred on *θ*_0_. If *E*(*θ*) *< E*(*θ*_0_), the new *θ *is automatically accepted and becomes *θ*_1_. In contrast, if *E*(*θ*) *> E*(*θ*_0_), *θ *is accepted with a probability exp [-*β *(*E*(*θ*) - *E*(*θ*_0_))], in which case it becomes *θ*_1_. If *θ *is rejected, then *θ*_1 _= *θ*_0_. This scheme is repeated for a predefined number of iterations *n*.

After *n *iterations the algorithm determines whether OEAMC sampling must stop. To do so, the viable parameter points found so far are divided into a predefined number of clusters. Then, OEAMC calculates the ellipsoids with minimum volume that enclose the points in each cluster and computes the sum of all ellipsoids volumes. The algorithm stops when the volume of all ellipsoids converges or when a maximum number of iterations is reached. If either of these criteria are met, OEAMC sampling terminates. Otherwise, *n *more iterations are carried out after updating *β *and ∑ according to

(6) $$\begin{array}{c} \beta =\left\{\begin{array}{cc} b\beta , & \text{if}\;f_{v}=0,\\ \beta , & \text{if}\;0<f_{v}\leq f_{0},\\ \beta ∕b, & \text{if}\;f_{v}>f_{0}, \end{array}\right)\\ \Sigma =\left\{\begin{array}{cc} s\Sigma , & \text{if}\;f_{a}>f_{u}\\ \Sigma , & \text{if}\;f_{l}<f_{a}\leq f_{u},\\ \Sigma ∕s, & \text{if}\;f_{a}<f_{l}, \end{array}\right) \end{array}$$

where *f_v _*and *f_a _*are the proportions of sampled viable parameter points and accepted transitions calculated over the last *n *iterations, respectively. The parameters *b*, *s *are larger than one and must be specified by the user. Equation (6) implies the following procedure. When Monte Carlo sampling is mainly confined to a viable region (*f_v _> f*_0_), *β *decreases and the frequency of accepted transitions increases. If this makes the frequency of accepted transitions larger than an upper limit (*f_a _> f_u_*), the covariance matrix ∑ will become larger and the method will sample broader regions. In contrast, when the method has not found any viable parameter point (*f_v _*= 0), *β *increases and the frequency of accepted transitions decreases in order to force the algorithm to sample regions with lower cost function. If this frequency falls below a lower limit (*f_a _< f_l_*), ∑ decreases to maintain the desired frequency of accepted transitions. The end product of OEAMC is the set *V_MC _*of all the viable parameter points that it found.

Several differences of OEAMC to existing approaches are worth noting. First, OEAMC does not increase *β *continuously from values near zero to values much larger than the maximum of the cost function, as in simulated annealing (see [42,43] and references therein). Furthermore, OEAMC does not utilize *β *as an "extra" stochastic parameter, an idea used in tempering approaches (see [44,45]). In addition, it does not diminish the adaptation of ∑ over time, as equilibrium adaptive Monte Carlo sampling does (see [45,46] and references therein). In contrast, OEAMC automatically adapts both *β *and ∑ during the whole sampling in order to obtain high and low frequencies of accepted transitions and viable parameter points, respectively. The objective of OEAMC is not to find a point close to the global optimum of the cost function, as in the case of simulated annealing, or to obtain a Markov chain with a specified equilibrium distribution, as in the case of equilibrium adaptive Monte Carlo sampling or simulated tempering. Instead, it aims to acquire a (potentially biased) sample of parameter points distributed all over the viable space.

### Multiple ellipsoid-based sampling

**Figure 3:**
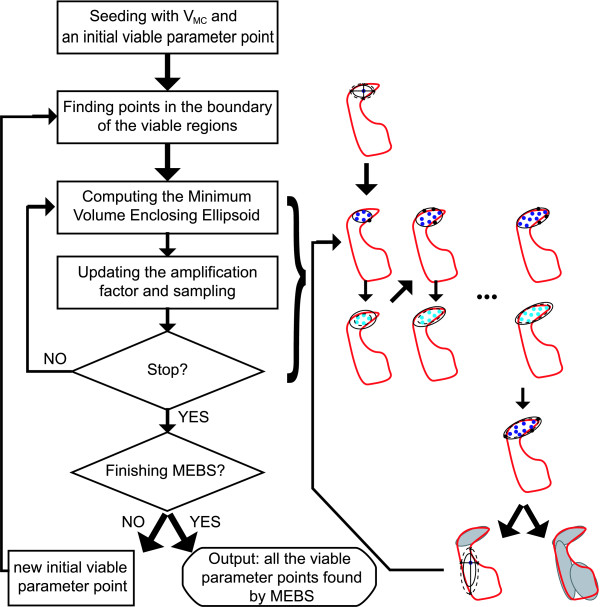
**Flowchart for the multiple ellipsoid-based sampling (MEBS) procedure**. Given *V_MC_*, the set of viable parameter points found by OEAMC, and an initial viable parameter point, the method finds viable parameter points near the boundary of the viable region. Then, it calculates the minimum volume enclosing ellipsoid (MVEE) that encloses those viable parameter points and samples inside an ellipsoid with the same orientation but smaller axes. In the figure, the ellipsoids inside of which sampling is carried out are represented by solid curves; dark blue and black points correspond to viable and nonviable points found in the last sampling, respectively; the MVEE ellipsoids are represented by dashed curves. After the sampling step just desribed, the method again calculates the MVEE of the viable points found so far (light blue points in the figure), and samples inside a scaled ellipsoid with the same orientation but larger axes (7). If the scaling factor tends to one, or a fixed number of iterations is reached, the initial exploration finishes. If this does not happen the method calculates the MVEE of the viable parameter points found and performs a new uniform sampling inside a new scaled ellipsoid. At the end of every new ellipsoid expansion, the algorithm checks if MEBS must stop, which occurs if the algorithm does not find any new viable points in viable nonexplored regions (grey ellipsoids). If MEBS does not stop, it carries out another ellipsoid expansion starting from a different viable parameter point. The result of the MEBS is the set of the viable parameter points found during all the ellipsoid expansions.

The OEAMC samples the viable space at low resolution. Thus, it is necessary to introduce a method that uses the viable points already found by OEAMC to explore the viable space in detail. A novel method we call multiple ellipsoid based sampling (MEBS) (Figure 3) carries out this fine-grained exploration of the viable space.

The use of an ellipsoid to bound viable regions in search spaces has been known for decades (see [47] and references therein). However, nonconvex viable regions are not accurately bounded by a single ellipsoid [48]. The problem is specially difficult in high dimensional spaces, where the "curse of dimensionality" forces the volume of the bounding ellipsoid to be much larger than the volume of the nonconvex bounded object of interest. The probability of "hitting" this object by sampling uniformly inside a bounding ellipsoid becomes negligible as the number of dimensions increases. To overcome this problem, MEBS iteratively constructs ellipsoids that start firstly from viable points already found by OEAMC, and then also by points found by MEBS. These ellipsoids change their centres and orientations in order to enclose multiple nearly convex viable regions and to cover the whole viable space as tightly as possible.

The *j*-th ellipsoid expansion starts by selecting a viable parameter point *θ*_*v*,*j *_in an adaptive way (see the Additional File 1 for details). In the first ellipsoid expansions the starting point will typically be a viable point obtained from OEAMC. This point defines 2*d *(*d *denotes the dimension of the parameter space) viable parameter points that are placed near the intersection between the boundary of the viable region and the straight lines parallel to the axes of the Cartesian coordinate system that pass through *θ*_*v*,*j *_(see Additional File 1 for a more detailed description). Then MEBS constructs an ellipsoid $L_{j}^{i}$. If *i *= 0, $L_{j}^{0}$ is the minimum volume ellipsoid that encloses the 2*d *viable points near the boundary of the viable space. If *i *≠ 0, $L_{j}^{i}$ is the minimum volume ellipsoid that encloses the set of viable points $V_{j}^{i}$ which comprises the viable points found after the iteration *i *of the *j*-th ellipsoid expansion. From this ellipsoid $L_{j}^{i}$, the MEBS creates a new ellipsoid $S_{j}^{i}$ that has the same orientation as $L_{j}^{i}$, but the lengths of its axes are multiplied by a scaling parameter *g_i_*. Then the algorithm uniformly samples a predefined number of parameter points *n *from this ellipsoid $S_{j}^{i}$. The union of the set of viable points in $S_{j}^{i}$ with $V_{j}^{i}$ then gives $V_{j}^{i+1}$.

The selection of the scaling parameter *g_i _*is critical for the performance of the algorithm. We define it as:

(7) $$g_{i}=\left\{\begin{array}{cc} g_{0}<1, & \text{if}\;i=0\\ g_{1}>1, & \text{if}\;i=1,\\ g_{i-1}+\frac{\left(g_{i-1}-1\right)}{p}, & \text{if}\;\left|V_{j}^{i}\right|-\left|V_{j}^{i-1}\right|>nb_{u},i>1,\\ g_{i-1}-\frac{\left(g_{i-1}-1\right)}{p}, & \text{if}\;\left|V_{j}^{i}\right|-\left|V_{j}^{i-1}\right|<nb_{l},i>1,\\ g_{i-1}, & \text{otherwise}\text{.} \end{array}\right)$$

where $V_{j}^{i}$ indicates the number of elements in the set and *b_l_*, *b_u_*, and *p <*1 are parameters for lower and upper bounds, and for axis scaling, respectively.

The rationale behind equation (7) is as follows: Points in $L_{j}^{0}$ lie near the boundary of the viable space. In high dimensional spaces the "curse of dimensionality" may cause a large proportion of this ellipsoid volume to be filled by nonviable points. Setting *g*_0 _*<*1 forces $S_{j}^{0}$ to be smaller than $L_{j}^{0}$. This makes it more likely that $S_{j}^{0}$ contains a larger proportion of viable parameter points, which will lead to a larger set $V_{j}^{0}$. To explore a larger elliptic region around *θ*_*v*,*j*_, the method then performs a second iteration with *g*_1 _*>*1. All subsequent iterations depend on the number of viable points found in the last iteration $\left(\left|V_{j}^{i}\right|-\left|V_{j}^{i-1}\right|\right)$. Specifically, when this number is larger than some upper limit *nb_u_*, the scaling parameter grows by a factor 1/*p >*1 to explore larger domains of parameter space. When the difference $\left(\left|V_{j}^{i}\right|-\left|V_{j}^{i-1}\right|\right)$ is below some lower limit *nb_l _*- only few additional viable points have been found in the last iteration - shrinking the axes allows an efficient exploration of smaller regions. Thus, viable parameter points found in previous iterations guide and define the ellipsoid where the next sampling is carried out.

The *j*-th ellipsoid expansion started from *θ*_*v*,*j *_finishes when *g_i _*converges to one or after a fixed number of iterations is reached. The output is *V*_*e*,*j*_, a set of sampled viable points that contains the 2*d *viable parameter points found near the boundary of the viable space, and the set of viable parameter vectors $\left|V_{j}^{i}\right|$ updated in the last iteration.

Then, the MEBS initiates a *j*+1-th ellipsoid expansion. The new initial point *θ*_*v*,*j*+1_, is chosen from the set composed by *V_MC _*and the union of *V*_*e*,*k*_, *k *= 1 ... *j*, that is, the set of viable points obtained after OEAMC exploration and previous ellipsoid expansions, respectively. To explore regions that have not yet been sampled, we preferentially select a *θ*_*v*,*j*+1 _that is far away from the average of all previous starting points *θ*_*v*,*k*_, *k *= 1 ... *j *(see Additional File 1 for details).

At the end of each ellipsoid expansion, the algorithm determines if MEBS should stop. To do so, the viable parameter points found so far {*V_MC_*, *V*_*e*,1_, *V*_*e*,2 _..., *V*_*e*,*j*_, *V*_*e*,*j*+1_} are divided into a predefined number of clusters. Then, MEBS calculates the ellipsoids with minimum volume that enclose the points grouped in each cluster and computes the sum of all ellipsoids volumes. The algorithm stops when the sum of the volume of all ellipsoids converges, or when a maximum number of ellipsoid expansions is reached. The final result of MEBS is the set of viable parameter points {*V_MC_*, *V*_*e*,1_, *V*_*e*,2_, ..., *V*_*e*,*j*_, *V*_*e*,*j*+1_}.

### Volume computation and acquisition of a large set of uniformly distributed viable parameter points

The end result of OEAMC and MEBS is a set of viable parameter points that can be used for a variety of purposes. Specifically, this set allows us to obtain simultaneously a large set of uniformly distributed viable points and an estimate of the viable volume Vol*_v_*. (Note that the set of viable points obtained by OEAMC and MEBS is not an uniform sample from the viable space).

To calculate Vol*_v _*we must evaluate the integral

(8) $$\begin{array}{c} \text{Vo}{\text{l}}_{v}=\int_{\Theta^{d}}f\left(\theta \right)d\theta ,\\ f\left(\theta \right)=\left\{\begin{array}{cc} 1, & \text{if}\;E\left(\theta \right)<E_{0},\\ 0, & \text{if}\;E\left(\theta \right)\geq E_{0}. \end{array}\right) \end{array}$$

Given *N *parameter points uniformly sampled in Θ*^d^*, the Monte Carlo integration theorem [49] implies that the volume (8) can be estimated by

(9) $$\begin{array}{c} \text{Vo}{\text{l}}_{v}=\int_{\Theta^{d}}f\left(\theta \right)d\theta ≃\text{Vo}{\text{l}}_{\Theta^{d}}\left\langle f\right\rangle ,\\ \left\langle f\right\rangle =\frac{1}{N}\overset{N}{\underset{i=1}{\sum }}f\left(\theta_{i}\right), \end{array}$$

where $\text{Vo}{\text{l}}_{\Theta^{d}}$ is the volume of the entire parameter space. If the error is Gaussian distributed, the standard deviation of the volume estimator is given by

(10) $$\begin{array}{c} \Delta \text{Vo}{\text{l}}_{v}=\text{Vo}{\text{l}}_{\Theta^{d}}\sqrt{\frac{\left\langle f^{2}\right\rangle -{\left\langle f\right\rangle }^{2}}{N}},\\ \left\langle f^{2}\right\rangle =\frac{1}{N}\overset{N}{\underset{i=1}{\sum }}f^{2}\left(\theta_{i}\right). \end{array}$$

Thus, if a high proportion of the *N *sampled parameter points is viable, the Monte Carlo integration in Θ*^d ^*will estimate the viable volume accurately.

This approach is usually sufficient to carry out viable volume estimations in low-dimensional spaces [8,21-25]. However, the "curse of dimensionality" poses a specific problem when this technique is applied to high-dimensional parameter spaces. To calculate the viable volume (9) and to obtain a large set of uniformly distributed viable parameters efficiently, one cannot simply sample over the entire parameter space, because doing so would be too inefficient. It would be much better to perform a uniform sampling over a subspace *W *∈ Θ*^d ^*that encloses the viable space as "tightly" as possible. This subspace will typically be much smaller than the entire space $\left(\text{Vo}{\text{l}}_{W}\ll \text{Vo}{\text{l}}_{\Theta^{d}}\right)$.

**Figure 4:**
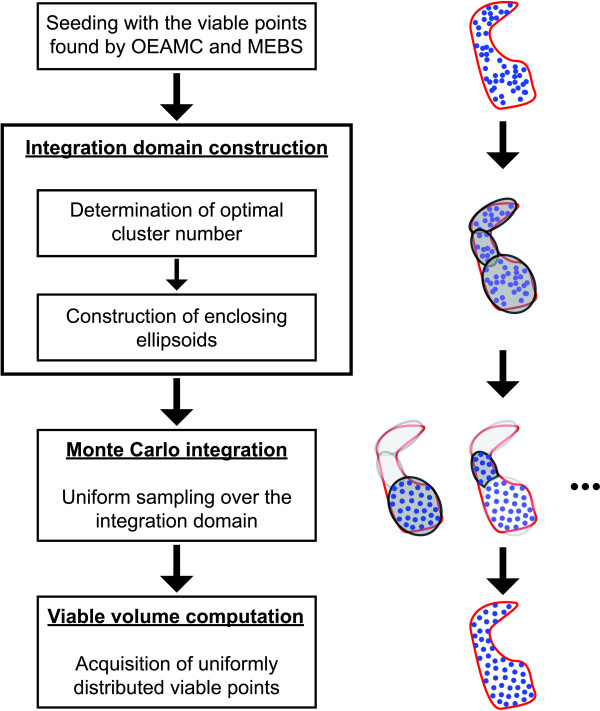
**Flowchart representing the algorithm for viable volume estimation, and the acquisition of a set of viable parameter points**. A set of viable parameter points found by OEAMC and MEBS (uppermost set of blue points in the figure) which are nonuniformly distributed over the whole viable space (area covered by the red curve in the figure) seeds the algorithm. Then, the method groups these points into *k *clusters (*k *= 3 in the hypothetical example shown), and calculates the ellipsoids with minimum volume that enclose the points in each cluster (11). After that, the algorithm performs a Monte Carlo integration of every ellipsoid (the intersections between ellipsoids are sampled only once) (12, 13). The result of the algorithm is a set of uniformly distributed viable parameter points (bottom set of blue points in the figure), from which the viable volume can be estimated.

To construct such a subspace (Figure 4), we build on the ideas already present in the algorithm developed by Hafner *et al. *[20]. The first step consists of using the set of viable parameter points *V_t _*that comprises the viable points already found by OEAMV and MEBS (the letter *t *stands for *total*). To make Vol*_v _*and Vol*_W _*as similar as possible, Hafner's method encloses the set of viable parameter points *V_t _*into a single box with a smaller volume than the entire space. However, in many dimensions the volume of a nonconvex viable space may be much smaller than the volume of its enclosing box. To overcome this limitation we define the subspace *W *via a family of ellipsoids that cover the viable space locally (do not confuse with the ellipsoid based exploration of the viable space described above). To determine these ellipsoids we group the set of viable parameter points *V_t _*into *k *clusters, and compute the ellipsoid with minimum volume that encloses the viable points grouped in every cluster (see Additional File 1 for details).

In this procedure, the subspace *W *is composed of the points of the parameter space enclosed by the *k *ellipsoids

(11) $$W=\left\{\theta \in \Theta^{d}\left|\theta \in ⋃W_{i}\right)\right\}\;i=1,2,\ldots ,k,$$

where *W_i _*is the region of the parameter space enclosed by the *i*-th ellipsoid. In general, the *k *ellipsoids may intersect, so the viable volume in *W *may be smaller than the sum of the viable volumes in *W_i_*. To avoid the resulting inaccuracy in volume estimation, we introduce a new integrand

(12) $$f_{i}\left(\theta \right)=\left\{\begin{array}{cc} 0, & \text{if}\;\theta \in ⋃W_{j},\;j=1,2,\ldots ,i-1,\\ 0, & \text{if}\;\theta \notin \Theta^{d},\\ f\left(\theta \right), & \text{otherwise}. \end{array}\right)$$

This integrand evaluates the parameter points in the ellipsoid intersections only once. Therefore, by sampling *N *parameter points points uniformly from *W *(11) and by using (9), we can estimate the viable volume (8) as

(13) $$\begin{array}{c} \text{Vo}{\text{l}}_{v}≃\int_{W}f\left(\theta \right)d\theta =\overset{k}{\underset{i=1}{\sum }}\int_{W_{i}}f_{i}\left(\theta \right)d\theta ≃\overset{k}{\underset{i=1}{\sum }}\text{Vo}{\text{l}}_{W_{i}}\left\langle f_{i}\right\rangle ,\\ \overset{k}{\underset{i=1}{\sum }}m_{i}=N, \end{array}$$

where *m_i _*is the number of parameter vectors sampled inside *W_i_*.

This approach of covering the viable region with ellipsoids can reduce the sampling volume dramatically, and thus increase the proportion of viable parameter points sampled in *W *far beyond that in the entire space Θ*^d^*. This means that the viable volume can be calculated more accurately, and larger sets of viable parameter points can be sampled uniformly.

We caution that in practice, one can never be certain that the whole viable space is contained in the integration domain *W *that our approach (or any other approach) determines. The agreement between the actual viable volume from expression (8) and the estimated viable volume (13) depends on the proportion of the viable volume that is enclosed in *W*. The subspace *W *is defined by the set of viable parameter points *V_t _*found by OEAMC and MEBS; therefore, the success of the volume estimation hinges on whether the previous exploration of parameter space found many viable points throughout the viable space. An implementation of our algorithm in MATLAB is available as the package HYPERSPACE from http://www.ieu.uzh.ch/wagner/software and http://www.csb.ethz.ch/tools/index.

## Results and Discussion

### A two-step algorithm for sampling of parameter spaces

The algorithm we propose starts from the definition of a viability condition and of a cost function (Figure 1). Depending on the biological model considered, the viability condition may include stability of a specific steady state, bistability [50], oscillations whose period lies in a given interval [20,24], the production of specific gene expression patterns [22], and many others. The cost function measures how closely the model's behavior matches the viability condition.

The first step of the algorithm consists of a global coarse-grained exploration of the viable space by an out-of-equilibrium adaptive Monte Carlo (OEAMC) sampling of the entire parameter space (Figure 2). Following a thermodynamic analogy used by simulated annealing [35] and Metropolis Monte Carlo sampling [36-41], we identify the parameter space and the cost function with the state space and the energy, respectively, of a thermodynamic system that is in contact with a thermal bath with variable temperature. The objective of OEAMC is to identify viable regions in the parameter space by adjusting the "temperature" and the length of the jumps through the parameter space. Briefly, OEAMC adapts the "temperature" and jump lengths to force a finite but small frequency of sampled viable parameter points, and a high proportion of accepted transitions to new parameter points. This helps OEAMC not to "get lost" in the parameter space, but at the same time lets it "travel" through nonviable regions where the cost function may have moderately high values. Thus, this procedure allows OEAMC to visit and sample from regions of the viable space that may be poorly connected to each other.

The low frequency of sampled viable parameter points forces OEAMC to explore the viable space at low resolution. To characterize the viable space in greater detail, it is necessary to define its borders more precisely, and to gain insight into its local geometry. In a second step, we therefore carry out a fine-grained exploration of the viable regions already identified through OEAMC, using a technique we call multiple ellipsoid-based sampling (MEBS) (Figure 3). This technique performs a local exploration of the parameter space by sampling from ellipsoids (an approach that is widely used in search algorithms, see [47] and references therein) that change their centres and expand or shrink their axes to enclose different regions of the viable space in which viable points are found. To cover locally nonconvex and/or poorly connected viable spaces, different ellipsoid expansions start from parameter points far away from each other (see Methods and Additional File 1).

The end result of OEAMC and MEBS is a set of viable parameter points that can be used for a variety of purposes. One of them is to define the integration domain in which a Monte Carlo integration estimates the volume of the viable space. (Note that the set of viable points obtained by OEAMC and MEBS is not an uniform sample from this space, and cannot be used directly for this purpose). We define this domain as the union of multiple ellipsoids - different from those used in MEBS sampling - that are constructed by grouping the viable parameter points into clusters, and by determining the ellipsoid with minimum volume that encloses the viable points in each of the clusters (Figure 4). This integration domain thus designed can cover nonconvex and high dimensional viable spaces "tightly". That is, the proportion of viable parameter points in this new integration domain is much higher than in the whole parameter space. By sampling viable points uniformly within this domain, we can compute the volume of a viable space. We reasoned that our procedure would allow us to reduce the computational effort in estimating a viable volume substantially. We will show in the next section that this is indeed the case. More generally, the large set of uniformly distributed viable parameter points that our method can generate permits us to characterize not only the size, but also the topology of a viable space. It also allows us to connect the robustness of a biological system to the geometrical properties of its viable space. Furthermore, this large set of viable parameters opens the possibility for a "glocal" analysis [20], in which the global characterization is supplemented by a local analysis around every viable parameter point. Thus, our algorithm can be used together with a local robustness measurement (e.g., that proposed by Dayarian *et al. *[7]) to get insight into the distribution of a model's robustness in a viable space.

### Efficient sampling of high-dimensional spaces

**Figure 5:**
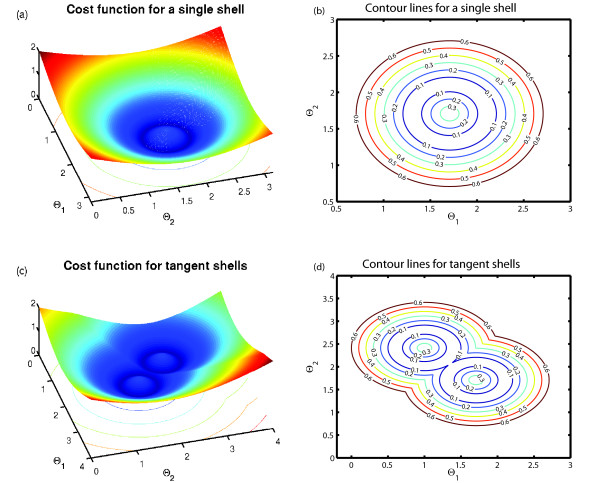
**Single and tangent spherical shells: cost function and viability condition**. The top-left and bottom-left panels show the cost function for a single and two tangent spherical shells, respectively, in a two-dimensional parameter space. The top-right and bottom-right panels show the contour plots that correspond to the left-side panels. In both cases, the viability condition is fulfilled by all the points enclosed by the two curves for which the value of the cost function is 0.1.

In a first test problem, we estimated the volume of a nonconvex region defined by either one single or two tangent multidimensional spherical shells (Figure 5). We chose this study system to analyze the efficiency of our method as a function of the geometry and dimension of a viable space, because here the viable volume can be calculated analytically.

We define the parameter space as Θ*^d ^*= Θ_1 _× Θ_2 _× ⋯ × Θ*_d_*, where Θ*_i _*= [-10, 10], *i *= 1, 2, ..., *d*. The cost function and the viability condition are given by

(14) $$\begin{array}{c} E_{n}\left(\theta \right)=\text{mi}{\text{n}}_{j}\left|||\theta -c_{j}||-\frac{r_{e}+r_{i}}{2}\right|,\;E_{n}\leq \frac{r_{e}-r_{i}}{2},\\ j=1,2,\ldots ,n,\;||c_{j}-c_{j-1}||=2r_{e}, \end{array}$$

where *c_j _*is a point in Θ*^d ^*and *r_e _*and *r_i _*are two scalars that fulfill *r_e _> r_i _*(in all our numerical tests *r_e _*= 0.5 and *r_i _*= 0.3).

When *n *= 1 (single spherical shell test case), the lines of constant cost are multidimensional spheres centred on *c*_1 _(Figure 5-b). The (degenerate) global minimum of the cost function occurs in the multidimensional sphere centred on *c*, and with radius $\frac{r_{e}+r_{i}}{2}$ (Figure 5-a, b). The viability condition is fulfilled by the parameter points that lie in the region enclosed by two multidimensional spheres with centre *c *and radii *r_i _*and *r_e_*, respectively.

For *n *= 2 (two tangent spherical shells test case), the cost function has its degenerate global minimum in two multidimensional spheres centered on *c*_1 _and *c*_2_, respectively, with radius $\frac{r_{e}+r_{i}}{2}$ (Figure 5-c, d); the viable parameter points lie in the inner region of two tangent multidimensional spherical shells with internal radii *r_i_*, external radii *r_e _*and centers *c*_1 _and *c*_2_, respectively.

The volume filled by the viable region can be computed analytically as:

(15) $$\begin{array}{c} \text{Vo}{\text{l}}_{v,t}=nC_{d}\left(r_{e}^{d}-r_{i}^{d}\right),\\ C_{d}=\left\{\begin{array}{cc} 1, & \text{if}\;d=0,\\ 2, & \text{if}\;d=1,\\ \frac{2\pi }{d}C_{d-2}, & \text{otherwise}, \end{array}\right) \end{array}$$

where *C_d _*is the volume of a *d *- dimensional hypersphere with radius 1.

**Figure 6:**
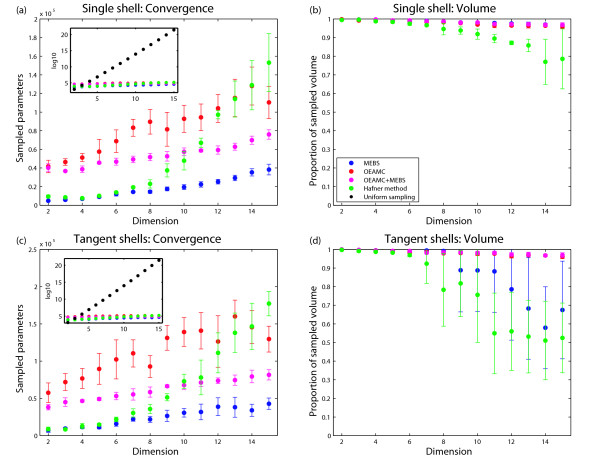
**Sampling efficiency of the single and tangent spherical shells test cases**. Panel and inset (a): Number of sampled parameters before convergence as a function of the dimension of the parameter space for the single spherical shell test case. The main panel and the inset show linear and logarithmic scales, respectively. Panel (b): Proportion of sampled viable volume before convergence for the single spherical shell test case. Panel and inset (c): Number of sampled parameters before convergence as a function of the dimension of the parameter space for the two tangent spherical shells test case. The main panel and the inset show linear and logarithmic scales, respectively. Panel (d): Proportion of identified viable volume before convergence for the two tangent spherical shells test case. Red, blue, magenta, green, and black circles represent the results obtained by OEAMC, MEBS, the combination of OEAMC and MEBS, the Hafner's method [20], and uniform samplings over the whole parameter space, respectively.

We now compare the performance of (i) MEBS and OEAMC alone, (ii) both of them together, (iii) uniform sampling, and (iv) the method proposed by Hafner *et al. *[20] based on Gaussian sampling (see the Additional File 1 for details). For the single spherical shell test case, MEBS and OEAMC alone, and the combination of both methods can identify the viable regions and obtain a good estimate of the viable volumes for dimensions up to *d *= 15 (Figure 6-b). Specifically, for all dimensions we studied they sample more than 95 per cent of the whole viable volume before converging. In addition, for this test case MEBS alone is much more efficient than OEAMC or a combination of both (Figure 6-a). Specifically, MEBS converges after sampling substantially fewer parameter points, because the frequency of viable points sampled by OEAMC is comparatively small, and OEAMC thus needs more sampling to estimate the viable volume to a given accuracy. For example, to achieve the same accuracy of volume estimation in *d *= 15 dimensions, MEBS uses 3-fold less samples than the OEAMC, and 2-fold less samples than the combination of both methods. In this first test case, the viable space, albeit nonconvex, is well-connected. This permits a ready exploration of the space by ellipsoid expansions - efficient "travel" of ellipsoids inside the viable volume is possible.

MEBS, OEAMC, and their combination are much more efficient than uniform sampling of the parameter space. For instance, at *d *= 15 dimensions, "brute force" sampling uses 17 orders of magnitude more sampling points to estimate the viable volume (Figure 6-a inset).

The Gaussian sampling carried out by Hafner's method *et al. *does not permit to identify in detail the borders of the viable volume for high dimensional spaces. Therefore, this technique can not estimate viable volumes in high dimensional spaces with precision (Figure 6-b). Moreover, in high dimensional spaces the tiny proportion of the whole parameter space filled by the viable volume forces this technique to sample a large number of viable points before converging (Figure 6-a). For example in *d *= 15 dimensions, Hafner's method uses 4-fold more samples than MEBS and underestimates the viable volume by 25 percent. 

For the test case of two tangent spherical shells, MEBS and Hafner's method often fail to "find" half of the viable volume in high dimensions (Figure 6-d). For example, in 14 dimensions, only 25 percent of the explorations carried out by MEBS and Hafner's method find both shells. The two methods share the same limitation: the inability of sampling a point from the second shell, when starting from a random parameter point in the first shell. To find the second shell starting from the first shell, MEBS and Hafner's method must sample from an ellipsoid or from a Gaussian distribution, respectively, both of which must cover viable regions from both shells. However, both also include nonviable parameter points. In high dimensions the fraction of viable points becomes very small, and the probability of finding a viable point from the second shell is very low.

In contrast, OEAMC alone, and the combination of both OEAMC and MEBS sample the viable regions well (Figure 6-d). Specifically, for up to *d *= 15 dimensions, they estimate the viable volume with an error smaller than a 5 percent. Importantly, the combination of both OEAMC and MEBS is more efficient than OEAMC alone (Figure 6-c). For instance, to achieve the same accuracy of volume estimation in *d *= 15 dimensions, the combination of MEBS and OEAMC used approximately 2-fold smaller samples than the OEAMC alone (and 17 order of magnitude smaller samples than uniform sampling).

The key for the success of the combination of OEAMC and MEBS is the complementary nature of their individual strengths. OEAMC does not need many sampled points to find two poorly connected regions. For example, in our two shell test case, it always hit both shells before sampling 25000 parameter in *d *= 15 dimensions. However, its low frequency of sampled viable points forces it to sample excessively many parameter points in order to explore a viable region in detail. In contrast, the bottleneck for the MEBS procedure is the discovery of a viable region - the second spherical shell in our example - that is poorly connected to a region that it already explored. Once such a region has been discovered by OEAMC, MEBS is able to sample from it efficiently, even if the region is nonconvex.

In sum, the combination of OEAMC and MEBS explores nonconvex and poorly connected viable regions in high dimensional parameter spaces more efficiently and accurately than either method alone and than other methods we evaluated. In addition, for both test cases the number of parameter points sampled by the combination of OEAMC and MEBS scales linearly with the number of dimensions (Figure 6-a and Figure 6-c). This suggests that for a given fixed complexity of the viable space, the computational effort needed by our method scales linearly with the dimensionality of the parameter space. This property makes our method suitable to explore high dimensional viable spaces.

### Model of a biochemical oscillator with two feedback loops

The viable space of a realistic model of a biological system is in general unknown. Therefore, it is necessary to get an estimate of the viable volume through uniform sampling in order to check the performance of our method. However, complex models may have tiny and complex viable spaces that make it infeasible to get such an estimate. This hampers the use of biological models with realistic complexity to characterize our algorithm. To illustrate the application of our method and to check its performance with a biological model, we therefore used a very simplified biological model containing only 12 parameters that permits us to compare the results of our method with the uniform sampling of the parameter space.

**Figure 7:**
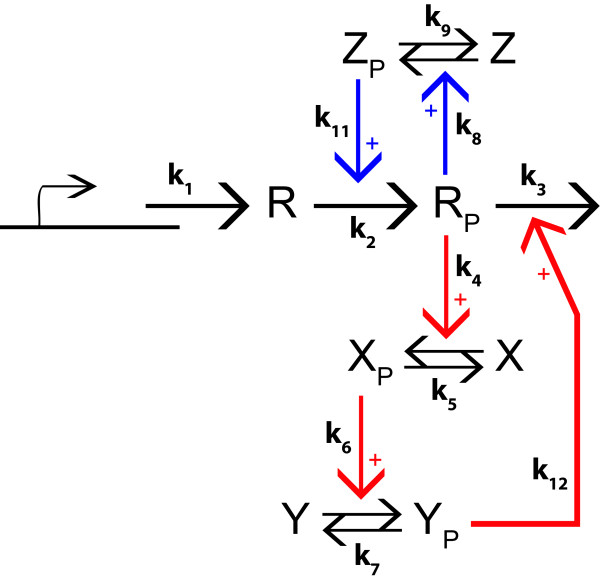
**Reaction diagram of the model of a simplified biochemical oscillator with two feedback loops proposed by Hafner *et al. ***[51]. The protein *R *is produced at a constant rate *k*_1 _and its phosphorylated state *R_p _*is produced at a rate, *k*_2_. The phosphorylated protein *Z_p _*modulates this phosphorylation rate by means of a positive feedback loop (blue diagram in the figure). In addition, *R_p _*is degraded with a rate, *k*_3 _that depends on the phosphorylated protein *Y_p _*by means of a negative feedback loop (red diagram in the figure).

This model describes a biochemical oscillator introduced by Hafner *et al. *[51]. It mimics the basic architecture of biological oscillators, such as cardiac pacemaker cells [52], intracellular calcium oscillations [53], cell cycle [27,54], and circadian clocks [55]. The model comprises two feedback loops (Figure 7) and it contains 12 individual parameters and 5 state variables which correspond to the concentrations of different proteins. Briefly, in this model a protein *R *is expressed, phosphorylated and degraded. Protein *R *can also auto-phosphorylate. In the positive feedback loop, the phosphorylated form *R_p _*acts as a kinase for protein *Z *whose active state *Z_p _*increases the auto-phosphorylation rate of *R*. This kind of positive loop is a basic mechanism behind substrate-depletion oscillators. An example is the maturation promoting factor (MPF) oscillator involved in the cell division cycle of frog eggs [56]. The negative feedback loop is composed of three steps: *R_p _*acts as kinase for an intermediate protein *X*. Its phosphorylated form *X_p _*phosphorylates a second protein *Y*, whose phosphorylated state *Y_p _*increases the degradation rate of *R*. Such negative feedback has been proposed as a basis for oscillations in many biological systems (see [27,28] for reviews).

The dynamics of the concentrations of the proteins *R *and *R_p _*follow mass action kinetics [57]

(16) $$\begin{array}{l} {}[\dot{R}]={\tilde{k}}_{1}-p\left(\left[Z_{p}\right]\right)[R],\\ {}[{\dot{R}}_{p}]=p\left(\left[Z_{p}\right]\right)[R]-n\left(\left[Y_{p}\right]\right)\left[R_{p}\right], \end{array}$$

where *p *([*Z_p_*]) and *n *([*Y_p_*]) respectively, reflect the effects of a positive and a negative feedbacks loops

(17) $$\begin{array}{c} p\left(\left[Z_{p}\right]\right)={\tilde{k}}_{2}+{\tilde{k}}_{11}\left[Z_{p}\right],\\ n\left(\left[Y_{p}\right]\right)={\tilde{k}}_{3}+{\tilde{k}}_{12}\left[Y_{p}\right]. \end{array}$$

In contrast, the concentrations of *X_p_*, *Y_p_*, and *Z_p _*are governed by Michaelis-Menten kinetics [57]

(18) $$\begin{array}{c} \left[{Ẋ}_{p}\right]=\frac{{\tilde{k}}_{4}\left[R_{p}\right]\left(\left[X_{T}\right]-\left[X_{p}\right]\right)}{{\tilde{k}}_{10}+\left(\left[X_{T}\right]-\left[X_{P}\right]\right)}-\frac{{\tilde{k}}_{5}\left[X_{P}\right]}{{\tilde{k}}_{10}+\left[X_{P}\right]},\\ \left[{Ẏ}_{p}\right]=\frac{{\tilde{k}}_{6}\left[X_{p}\right]\left(\left[Y_{T}\right]-\left[Y_{P}\right]\right)}{{\tilde{k}}_{10}+\left(\left[Y_{T}\right]-\left[Y_{P}\right]\right)}-\frac{{\tilde{k}}_{7}\left[Y_{P}\right]}{{\tilde{k}}_{10}+\left[Y_{P}\right]},\\ \left[{Ż}_{p}\right]=\frac{{\tilde{k}}_{8}\left[R_{p}\right]\left(\left[Z_{T}\right]-\left[Z_{P}\right]\right)}{{\tilde{k}}_{10}+\left(\left[Z_{T}\right]-\left[Z_{P}\right]\right)}-\frac{{\tilde{k}}_{9}\left[Z_{P}\right]}{{\tilde{k}}_{10}+\left[Z_{P}\right]}, \end{array}$$

where [*X_T _*], [*Y_T _*], and [*Z_T _*] denote the total concentration of *X*, *Y*, and *Z*, respectively. For the sake of simplicity, we normalize all concentrations to one, i.e., [*X_T_*] = [*Y_T_*] = [*Z_T_*] = 1.

The combination of active positive and negative feedback loops creates oscillators with a tunable frequency, and a robust amplitude [30]. These features make the negative plus positive loop oscillator suitable for systems like beating hearts and cell cycles. Here, we focused on oscillations in a narrow range of frequencies such as those produced by circadian clocks, and used the model to study the robustness of the oscillation period to parameter variations.

To explore broad ranges of parameters values we work in a logarithmic domain in which the logarithm of individual parameters are constrained as follows

(19) $$\begin{array}{c} k_{i}=\log\left({\tilde{k}}_{i}\right),\\ k_{i}\in \left[-4,2\right],\;\;i=1,2,\ldots ,10,\\ k_{i}\in \left[-7,2\right],\;\;i=11,12. \end{array}$$

Together, these ranges define the 12-dimensional parameter space Θ^12 ^= *k*_1 _× *k*_2 _× ⋯ × *k*_12_. We use the cost function

(20) $$E_{m}\left(\theta \right)=\left\{\begin{array}{cc} {\left[\left(T_{R_{p}}\left(\theta \right)-1\right)∕0.1\right]}^{2}, & \text{if}\;R_{p}\;\text{oscillates},\\ \infty , & \text{otherwise}, \end{array}\right)$$

where $T_{R_{p}}\left(\theta \right)$ is the period of the oscillations of *R_p _*for a parameter point *θ *= (*k*_1_, *k*_2_, ..., *k*_12_). The minimum of this cost function is attained by parameter vectors for which $T_{R_{p}}\left(\theta \right)=1$.

Finally, we introduced the viability condition

(21) $$E_{m}\leq 1,$$

meaning that a parameter point *θ *is viable if it causes *R_p _*to oscillate with a period in the narrow interval [0.9, 1.1].

**Figure 8:**
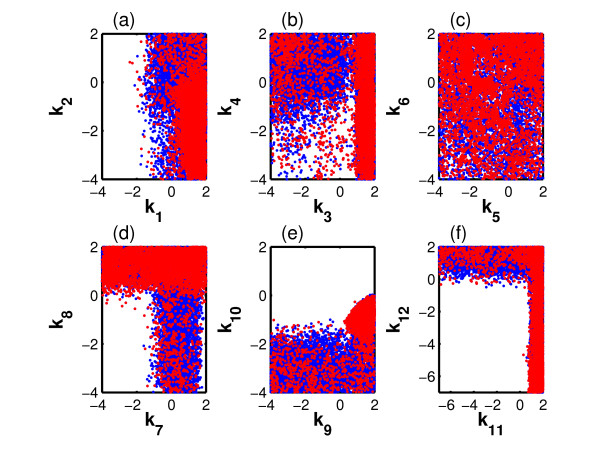
**Exploration of the viable space for the oscillator with two feedback loops**. Panels show projections of the 12-dimensional parameter space of the oscillator model onto six two-dimensional spaces corresponding to different parameter pairs. Red and blue points correspond to the viable parameter vectors found by OEAMC and MEBS, respectively.

To explore the viable space we carried out an OEAMC sampling followed by a MEBS. The viable parameter points obtained during this exploration are shown in Figure 8, which displays the 12-dimensional parameter space through six two-dimensional projections. The blue and red points, acquired by MEBS and OEAMC, respectively, occur in similar regions of the parameter space. This shows that the MEBS explored in detail the viable regions previously visited by OEAMC, just as for our spherical shells test case. The combination of OEAMC and MEBS revealed the nonconvexity of the viable space and its implications for the model function. Specifically, we note the viable region in Figure 8-f, which is composed of two approximately rectangular or bar-like regions that, together, form a nonconvex shape resembling an inverted L. Parts of these regions define topologies in which a single feedback loop produces the oscillations. More precisely, the left part of the horizontal bar corresponds to viable parameter points for which *k*_12 _is large and *k*_11 _small. In this region, only the negative feedback loop is active. Conversely, the bottom part of the vertical bar consists of viable parameter points for which *k*_12 _is small and *k*_11 _high. It corresponds to architectures where only the positive feedback loop is active (see Figure 7).

In a next step, we performed a Monte Carlo integration (see Methods and Additional File 1 for details) to estimate the viable volume. The integration domain is defined by using the viable points obtained by the OEAMC and MEBS explorations. This domain is approximately 630-times smaller than the whole parameter space. After uniformly sampling over the integration domain we obtained 3595 viable points, and estimated a viable volume of Vol*_v _*= 8.3 · 10^4 ^± 2 · 10^3^. To validate this estimate, we uniformly sampled over the whole parameter space with the same number of points we used in the OEAMC, MEBS, and integration parts of our algorithm. Only 9 of these points were viable, leading to a viable volume estimate of Vol*_v _*= 8.1 · 10^4 ^± 2.7 · 10^4^. The two estimates are very similar, but the estimation obtained through uniform sampling has an uncertainty one order of magnitude larger than the one calculated through our method. In addition, we uniformly sampled 4 · 10^7 ^points from the whole parameter space to compare the distributions of every single viable parameter. The results showed that the distributions of each of the 12 parameters obtained through our method and the extensive brute force sampling are very similar (Figure S1).

In sum, our method yields an accurate characterization of the viable space for this complex twelve-dimensional system at much higher efficiency than brute-force approaches. Specifically, by using the same number of sampling points it carries out a 13 times more accurate estimation of the viable volume, and obtains 400 times more uniformly distributed viable points.

### Robustness of positive and negative feedback loops

The sample of the viable space we obtained suggests a clear distinction between two oscillatory regimes, one driven by a positive and the other driven by a negative feedback loops. We next discuss these regimes, as an illustration of the type of analyses that our method enables.

The many viable parameter points we found allowed us to characterize key properties of model architectures with individual or combined feedback loops via the geometry of the viable space. For this purpose, we classified each of the viable points into one of the following categories:

• Essential negative feedback loop: The model keeps fulfiling the viability condition (21) after removing the positive loop, or after substituting this loop with a higher activation rate of *R_p _*(see Additional File 1).

• Essential positive feedback loop: The model keeps fulfiling the viability condition (21) after removing the negative loop or substituting this loop with a higher degradation rate of *R_p _*(see Additional File 1).

• Essential positive and negative feedback loops: No loop can be removed or substituted by a higher activation or degradation rate without violating the viability condition (21).

We found that model architectures for which the negative feedback loop is essential occupy the vast majority (86%) of the viable space we sampled. In contrast, significantly fewer parameter combinations lead to viable oscillations based on an essential positive loop (10%), or on a combination of essential positive and negative feedback loops (4%).

**Figure 9:**
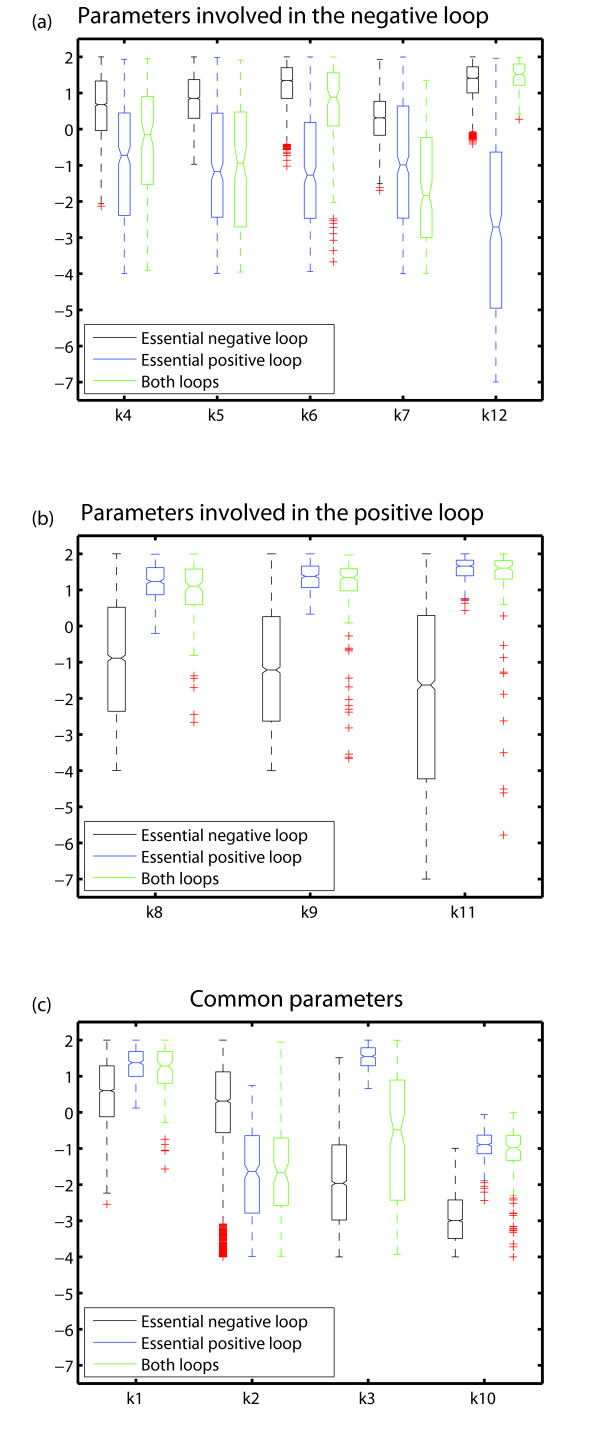
**Distribution of single parameters for model architectures with an essential negative, an essential positive, or essential positive and negative feedback loops**. The top, central, and bottom panels show the distribution of single parameters involved in the negative loop, positive loop, and not involved in any loop, respectively. Black, blue, and green boxplots correspond to parameter points that define architectures based on an essential negative loop, an essential positive loop, or essential positive and negative loop, respectively.

If a single loop is essential, the parameters mainly responsible for this loop will be constrained. These are parameters *k*_8_, *k*_9_, *k*_11 _for the positive loop, and parameters *k*_4_, *k*_5_, *k*_6_, *k*_7_, *k*_12 _for the negative loop (Figure 7). Figures 9-a and 9b illustrate these constraints. For example, in Figure 9-a, black coloring indicates to what extent parameters involved in the negative loop are constrained if this loop is essential, blue coloring indicates these constraints if only the positive loop is essential, and green coloring indicates these constraints if both loops are essential. Clearly, parameters involved in the negative loop can vary to a lesser extent if this loop is essential than when it is not essential. Analogous observations can be made for parameters involved in the positive loop (Figure 9-b).

A comparison of Figures 9-a and 9b also shows that parameters involved in the negative and positive feedback loops are constrained to different extents. Specifically, negative loop parameters can vary over broader intervals when the negative loop is essential than positive loop parameters can when this loop is essential. In addition, the parameters that do not form part of any loop (*k*_1_, *k*_2_, *k*_3_, *k*_10_) are more constrained in architectures with essential positive feedback loop than in topologies with an essential negative feedback loop (Figure 9-c).

Taken together, these observations imply that model architectures based on a negative loop fill more of the viable space, and allow individual parameters to vary more broadly than architectures based on positive feedback loops. In other words, model topologies based on an essential negative feedback loop are more robust than topologies with essential positive loops, or topologies with both essential positive and negative loops.

**Figure 10:**
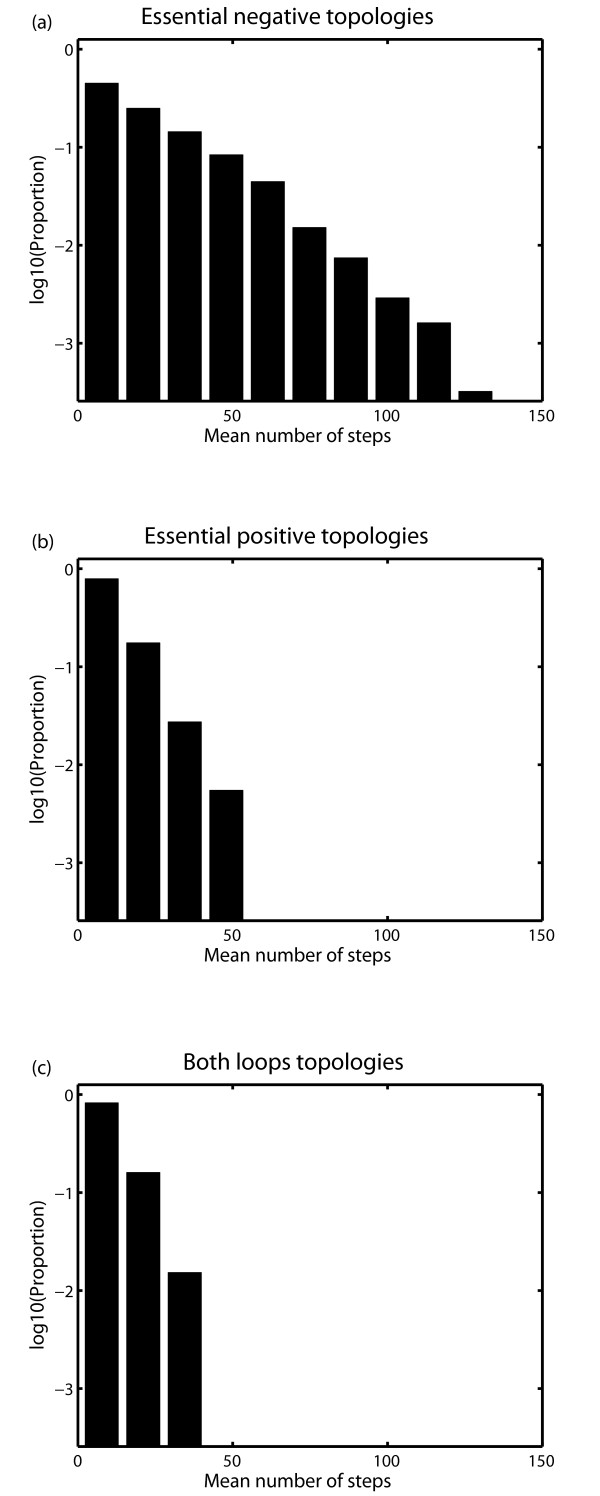
**Local robustness: distribution of the mean number of random walk steps needed to escape from the viable region for different model architectures**. Panels (a), (b), and (c) show the distributions of the mean number of steps for architectures based on essential negative, essential positive, as well as essential positive and negative feedback loops, respectively. The mean number of steps averaged over all the viable parameter points that define topologies with an essential negative feedback loop is significantly higher than the mean number of steps for oscillators with essential positive or a combination of negative and positive feedback loops (Wilcoxon rank sum test: *p*-value = 2.25 · 10^-29 ^and *p*-value = 4.0 · 10^-20^, respectively).

To further explore this aspect of robustness, we used the method proposed by Dayarian *et al. *[7] which estimates the number of steps that a random walk needs to escape from the viable space. Briefly, we started ten random walks from every viable parameter point. Each new point in a random walk was selected from an independent Gaussian distribution centred on the previous parameter point and with a diagonal covariance matrix with standard deviations *σ *= 0.01. We followed every random walk until it arrived at a nonviable parameter point, and recorded the number of steps it had taken to reach this nonviable point. We used this number of steps as an indicator of local robustness around such parameter point. The mean number of steps before exiting the viable region was higher if the starting point corresponded to an architecture with a negative loop than to an architecture with an essential positive loop, or to a combination of essential positive and negative loops (Figure 10). Moreover, the distribution of the number of steps for the negative feedback architectures has a long tail (Figure 10-a). Specifically, two times more steps may be needed to leave the viable space than for the other two architectures (Figure 10-b, c). Hence, also in terms of local properties revealed by this approach, architectures with an essential negative feedback loop are significantly more robust than other topologies.

**Figure 11:**
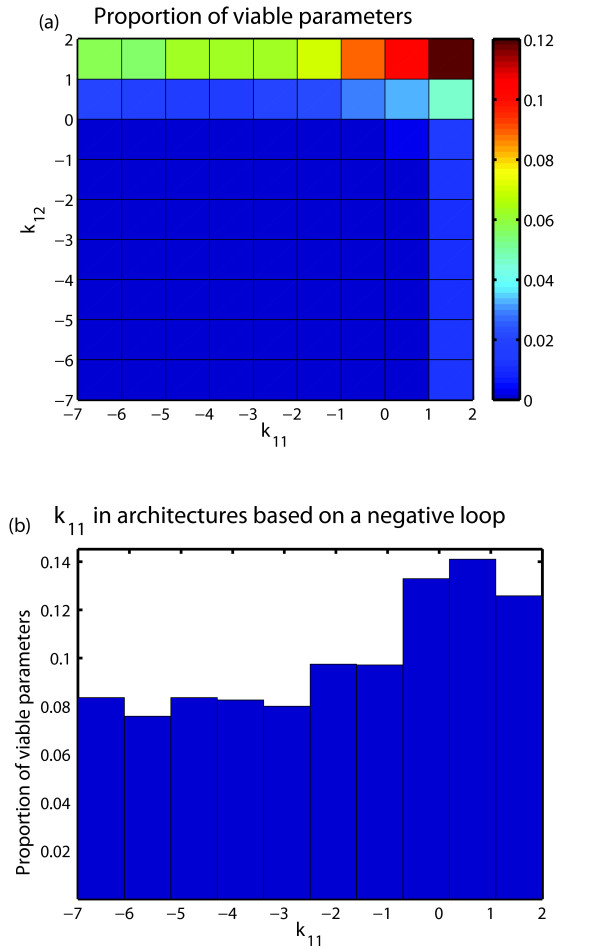
**Distribution of viable parameter points in the *k*_11 _*k*_12 _plane**. (a) proportion of viable parameter points found through Monte Carlo integration in every bin of the *k*_11 _*k*_12 _plane. The highest density of viable parameter points appears in configurations for which *k*_11 _and *k*_12 _are high; that is, model architectures in which both feedback loops are present (although one of them may not be essential). (b) proportion of viable parameter points which define architectures based on a negative feedback loop as a function of *k*_11_; that is, as a function of the single parameter that controls the strength of the positive feedback loop. The mean value of the parameter *k*_11 _is significantly higher (*p*-value = 2.0 · 10^-27 ^Wilcoxon signed rank test) than the centre of the interval in which *k*_11 _is defined. The density of viable parameter points increases with the value of the parameter *k*_11_.

In addition, we found that adding a positive (not necessarily essential) loop to a model architecture based on a negative feedback loop further increases robustness and the allowable range of parameter variation. Figure 11-a already hints at this observation, because it shows that the largest density of viable parameter points occurs in regions of parameter space where both *k*_11 _and *k*_12 _are high. These parameters are important for the positive and negative feedback loops, respectively. In regions with the most viable parameter points both feedback loops are active and at least one of these loops is essential.

Further analysis corroborates this observation. In architectures with an essential negative feedback loop, the mean value of the parameter *k*_11_, which controls the strength of the positive feedback loop, is significantly higher (*p*-value = 2.0 · 10^-27^; Wilcoxon signed rank test) than the centre of the interval in which *k*_11 _is defined. In other words, the randomly sampled architectures with an essential negative feedback loop preferentially occur in regions of parameter space where a positive loop is also active. Moreover, the density of viable parameter points increases with the value of the parameter *k*_11 _(Figure 11-b). Thus, a higher strength of the positive feedback loop increases the number of parameter combinations that gives rise to viable oscillations.

Taken together, these observations suggest that an added nonessential positive feedback loop gives a negative-loop-based model oscillator access to more viable parameter points. In the Additional File 1 we perform a similar analysis with a more complex model of a mammalian circadian oscillator. For this more realistic model we also observe that the circadian oscillations can be generated by a single negative feedback loop, whereas an additional positive feedback loop increases the robustness of the oscillations.

### Connectivity of the viable space

The connectivity of the viable space indicates to what extent different model architectures with the same behavior can change into one another through small changes in individual parameters, as might occur on evolutionary time scales.

To study this connectivity, we chose a set of viable points in which each of the three basic model architectures we consider are represented. For every pair of parameter points, we defined a straight line connecting them, and identified a set of three points that subdivide the line into four equally long segments (we also subdivided the line into 5, 6, 7, and 8 equally long segments, obtaining qualitatively identical results). We then asked whether each of these points was located in the viable space. If so, it may be possible to connect the two parameter points by a straight line that lies entirely in the viable space. Based on this information, we defined a graph whose nodes are the set viable parameter points. Two nodes are connected by an edge if the entire straight line between the nodes does not leave the viable space. Such an edge reflects the existence of potential evolutionary paths from one to the other node (parameter point) that does not leave the viable space. We find that this graph has one large connected component that comprises 95 percent of all nodes. This observation, together with our earlier analysis (Figure 8-f) shows that most of the viable space forms a nonconvex connected body with possible evolutionary trajectories that maintain the same behaviour and that connect qualitatively different system topologies through small changes in individual parameters.

The connected component contains nodes associated with all three basic architectures, but these three kinds of nodes are not equally likely to be connected to each other. Specifically, nodes (viable points) corresponding to model topologies with essential negative feedback loops are only connected to themselves, and to nodes with essential positive and negative feedback loops. Similarly, nodes that define topologies with essential positive feedback loops are only connected to themselves and to nodes with essential positive and negative feedback loops. Potential evolutionary trajectories that connect model architectures based on essential positive feedback loop and essential negative feedback loop, need to pass through configurations for which both loops are essential.

Overall, the global geometry of the viable space shows that model topologies based on an essential negative feedback loop are more robust than other architectures. Essential negative feedback allows the individual parameters to span larger intervals than essential positive feedback. Moreover, our local analysis reveals that topologies based on an essential negative feedback loop sustain the most change before losing viability. Successive small parameter changes can transform oscillators with an essential positive feedback loop into oscillators with an essential negative feedback loop, or vice versa. To do so, requires an intermediary stage in which both loops are essential.

## Conclusions

In biological systems, the diversity of biochemical parameter values that can lead to similar behavior makes it useful to introduce the concept of a viable space in which a biological system maintains a given function. The algorithm we present here allows an efficient exploration and characterization of such a viable space in systems with many parameters. It involves a global coarse grained identification of viable regions, followed by detailed local explorations of these regions. The global part of our algorithm can find viable regions that may be poorly connected. In the local part, the viable regions discovered in the global part are explored in detail. The exploration of the viable space allows us to identify a (typically nonconvex) subspace of the whole parameter space in which the proportion of viable parameter points is much higher than in the whole space. Knowledge of this subspace can dramatically reduce the number of samples needed to characterize the viable space. It also permits us to acquire a large number of uniformly distributed viable parameter points. The advantages of our method are especially dramatic in high-dimensional parameter spaces. It allows us to explore high dimensional nonconvex and poorly connected viable regions more efficiently and accurately than iterative Gaussian sampling [20] or uniform sampling of the entire parameter space [21-25]. Moreover, in the test problems we studied, the number of sampled parameters necessary to estimate the volume of the viable space to a given accuracy scales exponentially with the number of dimensions for Gaussian and uniform sampling, whereas it scales linearly for our algorithm. This suggests that for a given fixed complexity of the viable space, the computational effort of our method scales linearly with the dimensionality of the parameter space. This allows our method to explore high dimensional viable spaces efficiently.

An intrinsic limitation of our approach is imposed by the potential increase of the viable space's geometric complexity, when the dimension of the parameter space also increases. That is, increasing the dimensionality may cause the emergence of more poorly connected viable regions, which can exponentially increase the minimum number of iterations needed to identify all poorly connected viable regions and to sample them thoroughly. A second potential limitation concerns the identification of unconnected viable regions that are far from each other. The finite sampling frequency of viable parameter points required in the global exploration prevents one from "getting lost" in high dimensional spaces, but it may not allow the algorithm to travel across the wide nonviable region that may separates two viable regions far from each other. A third limitation includes that values for the parameters involved in the global and local explorations steps need to be chosen judiciously. These parameters include the maximum frequency of sampled viable points, bounds for the frequency of accepted iterations, and scaling factors for ellipsoid expansions.

Efficient sampling of the viable space allows one to accurately estimate the viable volume to assess model robustness, to study the topology of the viable space, and to carry out a "glocal" analysis [20], in which the global characterization of the viable space is supplemented by a local analysis. To illustrate how our method enables insights into the working of a biological system, we studied simple model of a biochemical oscillator with positive and negative feedback loops that involves 12 parameters [51]. We focused our attention on oscillations in a narrow range of frequencies such as those produced by circadian clocks, and used the model to study the robustness of the oscillation period to parameter variations. When characterizing the viable space composed by parameters for which the model oscillates in a narrow period interval, our method was 13 times more accurate in estimating the viable volume than uniform brute-force sampling. In addition, it obtained 400 times more uniformly distributed viable points.

We showed that the viable space of this oscillator forms a nonconvex connected body in which three classes of parameter points exist. They correspond to model architectures where the negative feedback loop, the positive feedback loop, or both loops are essential for fixed period oscillations. We also found that topologies with an essential negative feedback loop provide more robust fixed period oscillations than those based on an essential positive loop. Moreover, the addition of a nonessential positive feedback loop to a model with an essential negative feedback loop increases the number of parameter combinations that give rise to viable oscillations, and it therefore increases the robustness of fixed period oscillations. In spite of the model's simplicity, these results are consistent with well known structural properties of circadian oscillators: they typically rely on positive and negative feedback loops [58-60], the negative feedback alone is sufficient for fixed period oscillations [61-65], and the positive feedback loop increases the robustness of the oscillations to parameter changes [19,29-32]. These results reinforce the use of robustness as a tool for model discrimination [5,19]. Specifically, we observed that among the three model architectures that permit viable oscillations, the basic topology of circadian oscillators in nature coincides with the most robust one formed by an essential negative feedback loop and a non essential positive feedback loop. 

In summary, we have introduced an efficient algorithm that explores and characterizes the often tiny regions of a parameter space in which a model displays a desired behavior. We have applied our method to a biological model, but it is not restricted to such systems. It is suitable for all models with many parameters whose values are not well constrained by experimental data. Its spectrum of applications ranges from systems biology [66] all the way down to atomic physics [67].

An implementation of our algorithm in MATLAB is available as the package HYPERSPACE from http://www.ieu.uzh.ch/wagner/software and http://www.csb.ethz.ch/tools/index.

## Authors' contributions

Project planing: EZS, JS, AW. Development of the theory: EZS. Conceived and designed the experiments: EZS, MH, JS, AW. Performed the experiments: EZS. Analyzed the data: EZS, MH, JS, AW. Contributed reagents/materials/analysis tools: EZS, MH, AI. Creation of the figures: EZS, MH. Wrote the paper: EZS, JS, AW. All authors read and approved the final manuscript.

## Supplementary Material

Additional file 1 **Supplementary Information for "Efficient Characterization of High-Dimensional Parameter Spaces for Systems Biology"**. This document shows additional technical information about: • The calculation of minimum volume enclosing ellipsoids involved in OEAMC, MEBS, and the construction of the integration domain. • The determination of the number of clusters involved in the construction of the integration domain. • The acquisition of viable parameter points near the boundary of the viable space involved in the MEBS. • The choice of starting points for new ellipsoid expansions involved in MEBS. • The exploration and volume calculation of spherical shells. • The exploration exploration and volume calculation the viable space associated to biochemical oscillator model. • Characterization of the viable space of a model of the mammalian circadian oscillator with two feedback loops. Click here for file
