# Supplementary material for: Efficient characterization of high-dimensional parameter spaces for systems biology
Source: BMC Syst Biol. 2011 Sep 15;5:142. doi: 10.1186/1752-0509-5-142 (PMC3201035; doi:10.1186/1752-0509-5-142)
Supplement: Additional file 1 — Supplementary Information for "Efficient Characterization of High-Dimensional Parameter Spaces for Systems Biology". This document shows additional technical information about: • The calculation of minimum volume enclosing ellipsoids involved in OEAMC, MEBS, and the construction of the integration domain. • The determination of the number of clusters involved in the construction of the integration domain. • The acquisition of viable parameter points near the boundary of the viable space involved in the MEBS. • The choice of starting points for new ellipsoid expansions involved in MEBS. • The exploration and volume calculation of spherical shells. • The exploration exploration and volume calculation the viable space associated to biochemical oscillator model. • Characterization of the viable space of a model of the mammalian circadian oscillator with two feedback loops. [file 1752-0509-5-142-S1.PDF]

Supplementary Information for "Efficient Characterization of High-Dimensional Parameter Spaces for Systems Biology"

Elías Zamora Sillero*[1,2,3], Marc Hafner[1,3,4] , Ariane Ibig[2,3], Joerg Stelling[2,3]and Andreas Wagner[1,3,5,6]

[1]Department of Biochemistry, University of Zurich, Zurich, Switzerland
[2]Department of Biosystems Science and Engineering, ETH Zurich, Zurich, Switzerland
[3] Swiss Institute of Bioinformatics, Lausanne, Switzerland
[4] School of Computer and Communication, EPFL, Lausanne, Switzerland
[5] The Santa Fe Institute, Santa Fe, New Mexico, United States of America
[6] Department of Biology, University of New Mexico, Albuquerque, New Mexico, United States of America

Email: Elías Zamora Sillero*- e.zamora@bioc.uzh.ch; Marc Hafner -marc.hafner@epfl.ch ; Ariane Ibig -ariane.ibig@bsse.ethz.ch; Joerg Stelling - joerg.stelling@bsse.ethz.ch; Andreas Wagner - aw@bioc.uzh.ch ;

*Corresponding author

Supplementary Methods

Minimum volume enclosing ellipsoid calculation

The ellipsoid with minimum volume that encloses a set of N data points in a d-dimensional space can be constructed by solving the following optimization problem

$$\begin{cases} \text{Minimize,} & \log\left[\det\left(A^{-1}\right)\right], \quad \text{Vol} = v_0 \det\left(A^{-1}\right)^{\frac{1}{2}}, \\ \text{Subject to,} & (\theta_i - c)\, A\, (\theta_i - c)' \leq 1, \quad i = 1, 2, \ldots, N, \end{cases} \tag{1}$$

by means of a solver based on the Khachiyan algorithm [1], where A is the $d \times d$ matrix of the ellipsoid equation in the center form $(x - c) * A * (x - c)' = 1$, c is the center of the ellipsoid, and v_0 is the volume of the unit hypersphere in dimension d.

We estimated the runtime of this procedure as a function of the number of points in a data set (Figure S2.a). The results show a polynomial $O(N^{2.25})$ dependence on the number of points. The main effect of this polynomial complexity is to make the Monte Carlo integration computationally expensive, because it requires calculation of ellipsoids with minimum volume for large sets of viable parameter points involved in the definition of the integration domain.

To remedy this problem we developed a heuristic algorithm that approximates the ellipsoid obtained by the solver based on the Khachiyan algorithm, but requires much less time. Given a data set B_0 with N points from a d-dimensional space ($N \gg d$), the first step of this heuristic defines a set V_1 composed of n

points ($n \ll N$) that are randomly chosen from the initial data set. Then, it calculates the ellipsoid with minimum volume that encloses V_1 by using the solver based on the Khachiyan Algorithm, and obtains the ellipsoid matrix A_1, its center c_1, and its volume Vol_1. After that, the algorithm defines a new data set

$$B_1 = \left\{ \theta \in B_0 \left| (\theta - c_1) A_1 (\theta - c_1)' > 1 \right. \right\}, \tag{2}$$

that includes all the points in B_0 that were not enclosed by the ellipsoid defined by A_1 and c_1. In the second iteration the algorithm chooses n points randomly from B_1 and adds them to V_1 to form V_2. The algorithm then calculates the ellipsoid with minimum volume that encloses all the points in V_2, as well as its ellipsoid matrix A_2, its center c_2, and its volume Vol_2. The procedure then defines the data set B_2

$$B_2 = \left\{ \theta \in B_1 \left| (\theta - c_2) A_2 (\theta - c_2)' > 1 \right. \right\}, \tag{3}$$

which forms the basis for the next iteration. The algorithm stops when the ellipsoid volumes converge or when the set B_i is empty. The results of the algorithm are the matrix A_i, and the center c_i of the ellipsoid with minimum volume calculated in the last iteration before stopping.

By using the same data sets employed in the runtime test of the Khachiyan algorithm, we studied the complexity of our heuristic approach. The results show that, using $n = N/10$, the ellipsoid volumes estimated by the Khachiyan algorithm and our method are quite similar (Figure S2-b). Nevertheless, the complexity of our approach, albeit polynomial, has a much smaller exponent $O(N^{1.56})$ than the Khachiyan algorithm (Figure S2-a). In the analysis of the biochemical oscillator with two feedback loops, this property allowed us to calculate the ellipsoid with minimum volume that encloses the set of 19543 viable points found by MEBS and OEAMC approximately 1000 times faster than with the Khachiyan algorithm.

Construction of the integration domain: determination of the number of clusters

To calculate the viable volume and to obtain a large set of uniformly distributed viable parameters efficiently, one cannot simply sample over the entire parameter space, because doing so would be too inefficient. It would be much better to perform a uniform sampling over a subspace $W \in \Theta^d$ that encloses the viable space as ''tightly" as possible.

To construct such a subspace, we use the points found in the exploration steps (out of equilibrium adaptive Monte Carlo and multiple ellipsoid-based sampling) to obtain a large set of viable parameter points V_t. To define the subspace W we group this set into k clusters, and calculate the ellipsoids with minimum volume that enclose the viable parameter points grouped in every cluster. The integration domain is then defined by the union of all these ellipsoids.

We next explain the idea behind our heuristic clustering algorithm for cases where the viable space is not convex but could be well approximated by a set of n ellipsoid-like viable regions. In this case, a clustering algorithm should subdivide the space into $k = n$ clusters. Also in this case, n ellipsoids defined by n clusters will typically fill much less volume than $n-1$ ellipsoids defined by $n-1$ clusters (Figure S3), for the following reason. If the viable space has been subdivided only into $n-1$ clusters, at least one of the ellipsoids will typically enclose points from a non ellipsoid-like region, and much of its volume will be filled by non viable points. But when we use n ellipsoids, every ellipsoid will cover one ellipsoid-like region, and many nonviable parameter points will not be covered by any ellipsoid (Figure S3), rendering the volume covered by ellipsoids smaller.

When we use $n+1$ ellipsoids defined by $n+1$ clusters, one of the "old" n ellipsoids becomes subdivided into two ellipsoids placed in the same convex region (Figure S3). The total volume enclosed by the $n+1$-ellipsoids can not be much smaller than the volume filled by n-ellipsoids, because most of the "old" ellipsoid volume was already filled by viable parameter points (Figure S3).

In sum, if the viable space can be approximated by n-ellipsoids, the volume of these n-ellipsoids will typically be much smaller than the volume of $n-1$-ellipsoids that cover the viable space, but not much larger than the volume of $n+1$-ellipsoids. In order to choose a number of clusters similar to the number of ellipsoid-like regions of the parameter space we enforce this property. To do so, we found the following heuristic expression useful

$$k = \max_i \left(\frac{\mathrm{Vol}_{i+1}\mathrm{Vol}_{i-1}}{\mathrm{Vol}_i^2} \right), \quad \mathrm{Vol}_0 = \mathrm{Vol}_1. \tag{4}$$

Here, Vol_i is the sum of i ellipsoid volumes defined by grouping the viable points into i clusters. When the number of clusters increases, then each ellipsoid encloses fewer points, its mean axis length decreases, and the sum of the volume filled by the ellipsoids usually also decreases. That is, $\frac{\mathrm{Vol}_{i+1}}{\mathrm{Vol}_i} < 1$ will normally hold. According to expression (4), k is chosen such that Vol_k is much smaller than Vol_{k-1} but not much larger than Vol_{k+1}, just as we would desire from a clustering algorithm.

Finally, it is worth pointing out that the nonuniform distribution of the set of viable parameter points V_t does not allow the use of quality measures that emphasize homogeneity [2] to determine the number of clusters. The techniques used to explore the viable space and to obtain V_t (in our case OEAMC and MEBS) can sample some parts of the viable space in much more detail than others. This creates an artificially high concentration of viable points that is not proportional to the actual density of viable parameter points. Therefore, a method that emphasizes homogeneity would choose the number k of

clusters based on differences in the density of viable parameter points in V_t that are just artefacts of the exploration technique.

Acquisition of viable parameter points near the boundary of the viable space

In the beginning of every ellipsoid expansion, our MEBS method uses a bisection technique [3] to find $2d$ viable parameter points near the intersection between the boundary of the viable region and the straight lines parallel to the axes of the Cartesian coordinate system that pass trough the viable point $\theta_{v,i}$. These lines are defined as

$$r_i \equiv t\,\vec{e}_i + \theta_{v,i}, \quad i = 1, 2, \ldots, d. \quad t \in \mathbb{R}, \tag{5}$$

where $\vec{e}_i$ is a vector of length one parallel to the i-th axis and $\theta_{v,i}$ is the viable parameter point from which the i-th ellipsoid expansion starts.

The algorithm first determines whether the intersection point between the boundary of the parameter space and r_1 is viable. If so, this point is stored. If the point is not viable, the algorithm defines the following parameter points

$$a = \theta_{v,1}, \quad b = \Omega_\Theta \cap r_1, \quad t > 0, \tag{6}$$

where Ω_Θ stands for the boundary of the parameter space. Then, the algorithm determines whether the parameter point

$$c = \frac{b-a}{2}. \tag{7}$$

is viable. After that, it updates a and b

$$\begin{cases} a = c,\ b = b, & \text{if } c \text{ is viable,} \\ a = a,\ b = c, & \text{if } c \text{ is not viable,} \end{cases} \tag{8}$$

and calculates a new parameter point c from these updated values. It determines this points's viability, and continues iteratively in this manner until the Euclidean norm $\|b - a\|$ becomes smaller than a fixed threshold. At this point the parameter point a is saved as the final estimate of the intersection point between r_1 and the boundary of the viable region. The procedure is repeated for negative values of t, as well as for all other lines (axes) r_i $(i > 1)$. The result is a set of $2d$ viable parameter points that are close to the boundary of the viable region.

Choice of starting points for ellipsoid expansions

To be able to explore nonconvex viable spaces it is necessary to start ellipsoid expansions from viable parameter points placed in different regions of parameter space. Thus, after the first ellipsoid expansion

started from $\theta_{v,1}$, we must choose a new starting viable parameter point $\theta_{v,2}$ from the set composed by V_{MC} and $V_{e,1}$; that is, the set of viable parameter points obtained after the OEAMC exploration and the first ellipsoid expansion, respectively. We next explain how to choose $\theta_{v,2}$.

We choose the new starting parameter point preferentially far from the old starting point $\theta_{v,1}$, because we want to sample regions that have not yet been explored by MEBS. To do so, we first calculate the maximum and minimum distances from $\theta_{v,1}$ to all the viable parameter points included in V_{MC} and $V_{e,1}$

$$\begin{aligned} D_{max,1} &= \max_\theta \left\|\theta - \theta_{v,1}\right\|, \quad \theta \in \{V_{MC},\, V_{e,1}\}, \\ D_{min,1} &= \min_\theta \left\|\theta - \theta_{v,1}\right\|, \quad \theta \in \{V_{MC},\, V_{e,1}\}. \end{aligned} \tag{9}$$

Then, we introduce a stochastic variable D with probability density

$$\rho_1(D) = \begin{cases} 2\dfrac{D - D_{min,1}}{D_{max,1} - D_{min,1}}, & D \in [D_{min,1},\, D_{max,1}], \\ 0, & D \notin [D_{min,1},\, D_{max,1}]. \end{cases} \tag{10}$$

Thus, the stochastic variable D takes values close to $D_{max,1}$ with higher probability than values close to $D_{min,1}$.

Next, we sample a scalar D_1 from the distribution (10) and define the starting point $\theta_{v,2}$ for the new ellipsoid expansion as

$$\theta_{v,2} = \min_\theta \left(\left\|\theta - \theta_{v,1}\right\| - D_1\right), \quad \theta \in \{V_{MC},\, V_{e,1}\}. \tag{11}$$

The scalar D_1 has a high probability of being close to $D_{max,1}$; therefore, the starting point $\theta_{v,2}$ has a high probability of being far from $\theta_{v,1}$.

The next ellipsoid expansions follow an analogous principle. We calculate the maximum and minimum distances from all the viable points included in the set $\{V_{MC},\, V_{e,1},\, V_{e,2}, \ldots,\, V_{e,i}\}$ to the mean value of all the previous initial points

$$\begin{aligned} D_{max,i} &= \max_\theta \left\|\theta - \langle\theta_v\rangle\right\|, \quad \theta \in \{V_{MC},\, V_{e,1},\, V_{e,2}, \ldots,\, V_{e,i}\}, \\ D_{min,i} &= \min_\theta \left\|\theta - \langle\theta_v\rangle\right\|, \quad \theta \in \{V_{MC},\, V_{e,1},\, V_{e,2}, \ldots,\, V_{e,i}\}, \end{aligned} \tag{12}$$

where $\langle\theta_v\rangle$ is the mean value of $\{\theta_{v,1},\, \theta_{v,2}, \ldots,\, \theta_{v,i}\}$.

Once $D_{min,i}$ and $D_{max,i}$ are obtained, the stochastic variable D is redefined

$$\rho_i(D) = \begin{cases} 2\dfrac{D - D_{min,i}}{D_{max,i} - D_{min,i}}, & D \in [D_{min,i},\, D_{max,i}], \\ 0, & D \notin [D_{min,i},\, D_{max,i}]. \end{cases} \tag{13}$$

A scalar D_i sampled from the distribution (13) is used to define the starting point $\theta_{v,i+1}$ for the new ellipsoid expansion

$$\theta_{v,i+1} = \min_\theta \left(\|\theta - \langle\theta_v\rangle\| - D_i \right), \quad \theta \in \{V_{MC}, V_{e,1}, V_{e,2}, \ldots, V_{e,i}\}. \tag{14}$$

Because the scalar D_i has a high probability of being close to $D_{max,i}$, the starting point $\theta_{v,i+1}$ has a high probability of being far from $\langle\theta_v\rangle$.

Exploration and volume calculation of spherical shells: explanation of procedure

In the first test problem we sampled and calculated the volume of the viable region defined by either a single or two tangent spherical shells as a function of the dimension of the parameter space. The goal was to compare the performance of MEBS and OEAMC alone, with the combination of both methods, as well as with uniform sampling, and with a method proposed by Hafner *et al.* [4] based on Gaussian sampling. The OEAMC sampling started from a random viable point and used an updating interval of $n = 2000$, an upper limit of the frequency of viable sampled parameter points of $f_0 = 0.01$, as well as lower and upper bounds of the frequency of accepted transitions of $f_l = 0.2$ and $f_u = 0.3$, respectively. The initial covariance matrix Σ was diagonal with entries 0.02, and the updating factor was equal to $s = 1.5$. The initial β was equal to one and the corresponding updating factor equal to $b = 1.5$. Every 8000 iterations OEAMC grouped the viable points it had found so far into d different clusters (d denotes the dimensionality of the parameter space). Then it calculated the minimum volume ellipsoid that encloses the viable points in each cluster, and calculated the volume of the intersection of all ellipsoids through a Monte Carlo integration. The algorithm stopped when the estimated volumes differed for the last four iterations by less than 1 percent and 80 percent for the cases of OEAMC alone and OEAMC combined with MEBS, respectively. We used MEBS with 100 parameter points sampled inside every ellipsoid, scaling factors $g_0 = 0.3$, $g_1 = 2, p = 3$, and upper and lower bounds of the fraction of viable sampled parameters $b_u = 0.5$ and $b_l = 0.1$, respectively. In every iteration, before starting to expand ellipsoids from a new initial point, MEBS grouped the viable points found so far into d different clusters. Then it calculated the minimum volume ellipsoid that encloses the viable points in every cluster, and calculated the volume of the intersection of all of them. The algorithm stopped when the estimated volumes differed for the last four iterations by less than a specified value. Specifically, we required that the estimated volumes differed by less than 1 percent less both in the cases of the MEBS alone or in combination with OEAMC.

We applied the method of Hafner and coworkers starting from a random viable point. We used a maximum

number of $5 \cdot 10^4$ sampled parameter per iteration, and asked that $5 \cdot 10^2$ viable parameters had been identified to change to the next iteration. The parameters for the Gaussian samplings and the convergence conditions were equal to those published in [4].

To compare MEBS and OEAMC alone, the combination of both, and the method of Hafner *et al.*, we first enclosed all the viable points found by each method with an ellipsoid with minimum volume, in order to estimate the volume of the viable space. We then calculated this volume by using a Monte Carlo integration that sampled uniformly $1 \cdot 10^5$ parameter points over the minimum volume ellipsoid we had generated. Finally, to test the performance of "brute force" uniform sampling, we calculated analytically the mean number of sampled parameter points that an uniform sampling of the whole parameter space would need to find $2d + 1$ viable parameters in each spherical shell. (This points define a d-dimensional ellipsoid). This was done by considering every sampled parameter point as a sample from a binomial distribution with a probability of being viable equal to the ratio between the viable volume and the volume of the whole parameter space.

Two feedback loop oscillator: exploration of the parameter space and calculation of the viable volume

The cost function used to explore the parameter space of the two feedback loops model is given by

$$E_m(\theta) = \begin{cases} \left[(T_{R_p}(\vec{\theta}) - 1)/0.1\right]^2, & \text{if } R_p \text{ oscillates,} \\ \infty, & \text{otherwise,} \end{cases} \tag{15}$$

where $T_{R_p}(\theta)$ is the period of the oscillations of $[R_p]$ for a parameter point θ. The viability condition is $E_m \leq 1$.

Before performing an OEAMC exploration of the parameter space, it is necessary to take into account that the cost function (25) is unbounded for parameter points that do not allow oscillations. Thus, when the frequency of sampled viable parameters is larger than the upper bound, $f_v > f_0$, β decreases, but the frequency of accepted transitions may not increase because the cost function is not finite for a large proportion of parameter space. Similarly, the elements of Σ may not increase, and the method may not be able to sample increasingly broad regions of the parameter space. To eliminate this problem we modified our update rules as follows

$$\beta = \begin{cases} b\beta, & \text{if } f_v = 0, \\ \beta, & \text{if } 0 < f_v \leqslant f_0, \\ \beta/b, & \text{if } f_v > f_0, \end{cases} \qquad \Sigma = \begin{cases} \Sigma/s, & \text{if } f_v = 0, \\ \Sigma, & \text{if } 0 < f_v \leqslant f_0. \\ s\Sigma, & \text{if } f_v > f_0. \end{cases} \tag{16}$$

This modification may diminish the efficiency of OEAMC, because it does not impose a high number of accepted transitions, but it forces the elements of Σ to be larger when β decreases.

We carried out OEAMC sampling for the oscillator model beginning from a viable parameter point for which the negative feedback loop is inactive and the positive loop is active. Different viable initial points yielded similar results (not shown). The parameters of the OEAMC sampling were as follows: updating interval $n = 400$, upper limit of the frequency of viable sampled parameter points $f_0 = 0.02$, updating factors $s = 1.5$ and $b = 1.5$. The initial covariance matrix Σ was diagonal, with entries $\sigma_{i,i} = 0.06$ for $i = 1, 2, \ldots, 10$, and $\sigma_{i,i} = 0.09$ for $i = 11, 12$. The initial value of β was equal to 1.
After $2 \cdot 10^5$ iterations we obtained 8832 viable parameter points. We then grouped these viable points into 12 clusters (equal to the number of dimensions, but we obtained similar results using different numbers of clusters). We used the elements of each cluster as seeds to initialize 12 different parallel MEBS procedures with the following parameters: $n = 24$ points sampled inside every ellipsoid, scaling factors $g_0 = 0.3$, $g_1 = 2, p = 3$, upper and lower bounds of the fraction of viable sampled parameters $b_u = 0.5$ and $b_l = 0.1$, respectively. The result of the EBSs was a set of 10711 viable parameter points from 14470 sampled parameter points.
Last, we performed a Monte Carlo integration to estimate the viable volume. Following the heuristic (4), we grouped the viable parameter points into 3 clusters, which served to define the integration domain. This integration domain was approximately 630-times smaller than the whole parameter space. After uniformly sampling $3.34 \cdot 10^5$ parameter points from the integration domain, we obtained 3595 viable points and a viable volume $\mathrm{Vol}_v = 8.3 \cdot 10^4 \pm 2 \cdot 10^3$.
These viable points show that the different regions of viable space correspond to model architectures that involve an essential negative feedback loop, an essential positive feedback loop, or both. For every viable parameter point, we asked whether one of the two feedback loops was essential by removing it. To do so, we set all the parameters involved in the loop equal to zero and determined whether the system lost viability. If so, we checked if the role of the loop merely served to increase the activation rate (for a removed positive loop) or the degradation rate (for a removed negative loop) of R_p without being directly involved in the creation of sustained oscillations. To do so, we increased all along the parameter domain the value of k_2 (k_3) which control the activation (degradation) rate after having removed the positive (negative) loop, and determined whether doing so reinstated viability.

Model of the mammalian circadian oscillator with two feedback loops

The aim of this analysis is to show the feasibility of our method to explore the viable space of models with realistic complexity. We choose to study the model of the mammalian circadian oscillator proposed by

Becker-Weimann *et al.* [5], in order to complement the robustness analysis of the simplified biochemical oscillator model.

The model comprises two feedback loops (see Figures 1 and 2 in [5] for schematic diagrams of the model). It contains 24 individual parameters and 7 state variables that are governed by the following set of ODEs

$$\frac{dy1}{dt} = f(trans_{Per2/Cry}) - k_{1d}y1, \tag{17}$$

$$\frac{dy2}{dt} = k_{2b}y1^q - k_{2d}y2 - k_{2t}y2 + k_{3t}y3, \tag{18}$$

$$\frac{dy3}{dt} = k_{2t}y2 - k_{3t}y3 - k_{3d}y3, \tag{19}$$

$$\frac{dy4}{dt} = f(trans_{Bmal1}) - k_{4d}y4, \tag{20}$$

$$\frac{dy5}{dt} = k_{5b}y4 - k_{5d}y5 - k_{5t}y5 + k_{6t}y6, \tag{21}$$

$$\frac{dy6}{dt} = k_{5t}y5 - k_{5t}y6 - k_{5d}y6 + k_{7a}y7 - k_{6a}y6, \tag{22}$$

$$\frac{dy7}{dt} = k_{6a}y6 - k_{7a}y7 - k_{7d}y7. \tag{23}$$

Both transcription rates, $f(trans_{Per2/Cry})$ and $f(trans_{Bmal1})$, are described by Hill functions

$$f(trans_{Per2/Cry}) = \frac{v_{1b}(y7 + c)}{k_{1b}\left(1 + (y3/k_{1i})^p\right) + (y7 + c)}, \quad f(trans_{Bmal1}) = \frac{v_{4b}y3^r}{k_{4b}^r + y3^r}. \tag{24}$$

We briefly describe the key regulatory mechanism behind this model. An activated form of BMAL1 (BMAL1*, $y7$) promotes the transcription of Per2 and Cry genes, resulting in an increase of Per2/Cry mRNA ($y1$). As the levels of PER2 and CRY proteins increase, they form a complex ($y2$), which is transported into the nucleus. The nuclear PER2/CRY complex ($y3$) inhibits Per2/Cry transcription and closes the negative feedback loop. In a positive feedback loop, the PER2/CRY complex ($y3$) also activates Bmal1 transcription. Thus, Bmal1 mRNA ($y4$) and protein ($y5$) concentrations increase. As a result, nuclear BMAL1 ($y6$) in its active form (BMAL1*, $y7$) restarts transcription of Per2 and Cry genes.

To define the parameter space, the individual parameters q, r, and c are set equal to their values used in [5]. The remaining parameters are allowed to change by four orders of magnitude around their values used in [5] in a logarithmic scale. Taken together, this means that the parameter space to be explored has 21 dimensions.

We use the cost function

$$E_m(\theta) = \begin{cases} \left[(T_{y3}(\theta) - 24)/2.4\right]^2, & \text{if } y3 \text{ oscillates,} \\ \infty, & \text{otherwise,} \end{cases} \tag{25}$$

where $T_{y3}(\theta)$ is the period of the oscillations of the nuclear PER2/CRY complex ($y3$) for a given parameter point θ. The minimum of this cost function is attained by parameter vectors for which $T_{y3}(\theta) = 24$ hours.

Finally, we use the viability condition

$$E_m \leq 1, \tag{26}$$

meaning that a parameter point θ is viable if it makes the nuclear PER2/CRY complex ($y3$) oscillate with a period in the interval $[21.6\,h, 26.4\,h]$.

Robustness and characterization of the viable space

The transcription rates of Per2 and Cry genes are governed by a constant activator (dependent of the parameter c), as well as by a negative, and a positive feedback loops. The Becker-Weimann model can not oscillate without the negative feedback loop [5]. In contrast, for certain parameters it can carry out viable oscillations after deleting the positive feedback loop [5]. This contrasts with the observation that the mammalian circadian oscillator relies on both positive and negative feedback loops [6–8].

To analyse the robustness implications of the positive feedback loop and the constant activator, described by the parameter c, we have explored the viable spaces of three different models: a "full" model with both positive feedback loop and constant activator, a model with just a constant activator, and a model with only a positive feedback loop. For every model we first performed OEAMC sampling, followed by MEBS. In a next step, we performed a Monte Carlo integration to estimate the viable volume and to obtain a large set of uniformly distributed viable points. The integration domains are defined by using the viable points obtained in the OEAMC and MEBS sampling.

We then classified the uniformly sampled viable points found in the exploration of the full model into one of the following non exclusive categories:

- Rhythmic Δv_{4b}: The full model keeps fulfiling the viability condition (26) after removing the positive loop.
- Rhythmic Δc: The full model keeps fulfiling the viability condition (26) after removing the constant activator .
- Essential constant activator and positive feedback loop: Neither the positive feedback loop nor the constant activator can be removed without violating the viability condition (26).

We found that parameter points for which the positive feedback loop can be removed occupy the majority (87%) of the viable space. In contrast, substantially fewer parameter combinations (20%) lead to viable oscillations after deleting the constant activator. Only 5 % of the viable points have both an essential

constant activator and an essential positive feedback loop. In other words, the viable space is mainly filled by parameters for which the model retains viability after elimination of the positive feedback loop. A deletion of the constant activator frequently causes a loss of viability.

We compared the robustness of the full model and of the simpler models with just a constant activator or a positive feedback loop. To do so, we used the method proposed by Dayarian *et al.* [9] which estimates the number of steps that a random walk needs to escape from the viable space (see Methods). For each model, and starting from every viable parameter point that we had previously identified during the Monte Carlo integration, we determined the number of steps that the random walk needed to leave the viable space. We used this number of steps as an indicator of the oscillator's robustness to perturbations in such parameter points. The mean number of steps before exiting the viable region was significantly higher for the rhythmic Δv_{4b} full model than in the model with just a constant activator (Figure S4.a). Specifically, we observed that the robustness of the rhythmic Δv_{4b} full model is higher at all concentrations of BMAL1*(the concentration of BMAL1* is equal to the sum of the parameter c and the mean value of $y7$). These observations imply that adding a positive feedback loop to a circadian oscillator model that oscillates with just a constant activator increases the robustness of the model. In contrast, the mean number of steps to exit the viable region was not significantly higher for the rhythmic Δc full model than in the model with just a positive feedback loop (Figure S4.b). In other words, the addition of a constant activator to an oscillator with just a positive feedback loop does not increase its robustness. Lastly, a full model for which neither the positive feedback loop nor the constant activator can be removed is significantly less robust than the other two models (Figure S4.c). This implies that the addition of a positive feedback loop to a nonviable oscillator with just a constant activator can turn the model viable, but provides little robustness.

In summary, our analysis shows that a model of the mammalian circadian oscillator with higher complexity than the model we discussed in the main text shows similar features: parameters that define an oscillator's architecture with an essential negative feedback loop and a nonessential positive feedback loop fill the most part of the viable space. Moreover, this architecture improves the robustness of the model against changes in the parameter values (this property was previously observed in different models of circadian oscillators [10–14]). In addition, the analysis also shows that our method remains feasible for systems with more than 20 dimensions.

We finally point out that our observations for the Becker-Weimann model agree with the hypothesis formulated by Trane [14] which states that circadian oscillations are generated by a single negative feedback loop, whereas the additional positive feedback loop increases the robustness of the oscillations.

Acknowledgements

We would like to acknowledge support through SNF grants 315200-116814, 315200-119697, and 315230-129708, as well as through the YeastX project of SystemsX.ch.

References

1. Khachiyan L: **Rounding of polytopes in the real number model of computation**. *Mathematics of Operations Research* 1996, **21**:307–320.
2. Spath H: *Cluster Dissection and Analysis: Theory, FORTRAN Programs, Examples*. New York: Halsted Press 1985.
3. Press WH, Teukolsky SA, Vetterling WS, Flannery BP: *Numerical Recipes in C*. New York: Cambridge University Press 1992.
4. Hafner M, Koeppl H, Hasler M, Wagner A: **"Glocal" Robustness Analysis and Model Discrimination for Circadian Oscillators**. *PLoS Comput. Biol.* 2009, **5**:e1000534.
5. Becker-Weimann S, Wolf J, Herzel H, Kramer A: **Modeling Feedback loops of the Mammalian Circadian Oscillator**. *J. Biophys.* 2004, **87**:3023–3034.
6. Lee K, Loros J, Dunlap JC: **Interconnected feedback loops in the Neurospora circadian system**. *Science* 2000, **289**:107–110.
7. Gallego M, Virshup DM: **Post-translational modifications regulate the ticking of the circadian clock**. *Nat. Rev. Mol. Cell. Biol.* 2007, **8**:139–148.
8. Rust MJ, Markson JS, Lane WS, Lane DS, Fisher DS, O'Shea EK: **Ordered Phosphorylation Governs Oscillation of a Three-Protein Circadian Clock**. *Science* 2007, **318**:809–812.
9. Dayarian A, Chaves M, Sontag E, Sengupta A: **Shape, Size, and Robustness: Feasible Regions in the Parameter Space of Biochemical Networks**. *PLoS Comp. Biol.* 2009, **5**:e1000256.
10. Cheng P, Yang Y, Liu Y: **Interlocked feedback loops contribute to the robustness of the Neurospora circadian clock**. *Proc. Natl. Acad. Sci. USA.* 2001, **98**:7408–7413.
11. Tsai T, *et al*: **Robust, Tunable Biological Oscillations from Interlinked Positive and Negative Feedback Loops**. *Science* 2008, **321**:126–129.
12. Saithong T, Painter KJ, Millar AJ: **The Contributions of Interlocking Loops and Extensive Nonlinearity of Circadian Clock Models**. *PLoS ONE* 2010, **5**:e13867.
13. Saithong T, Painter KJ, Millar AJ: **Consistent Robustness Analysis (CRA) Identifies Biologically Relevant Properties of Regulatory Network Models**. *PLoS ONE* 2010, **5**:e15589.
14. Trane C: **Robustness Analysis of Intracellular Oscillators with Application to the Circadian Clock**. *PhD thesis*, Royal Institute of Technology, Automatic Control Lab 2009.
15. Eadie W, Drijard D, James F, Roos M, Sadoulet B: *Statistical Methods in Experimental Physics*. Amsterdam: North-Holland 1971.

Figures

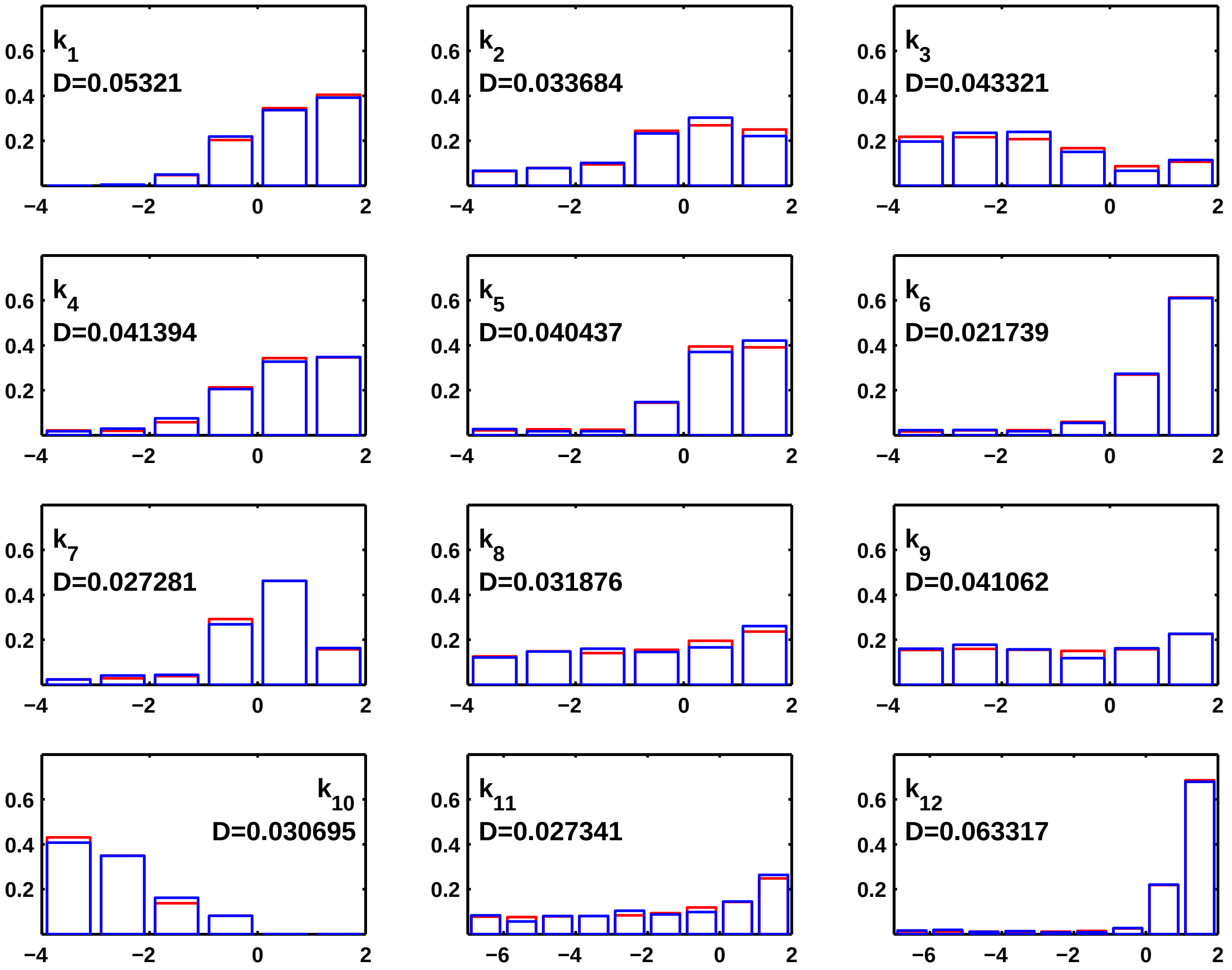

Figure 1. Distribution of every single parameter obtained through a "brute force" sampling (blue) and our method (red). The 694 viable points acquired through a "brute force" sampling where obtained by uniformly sampling $4 \cdot 10^7$ parameter points over the whole parameter space. The 3595 viable points acquired through our algorithm were obtained by checking $5.44 \cdot 10^5$ parameter points in both the exploration and integration steps. For every single parameter the distance D between both distributions is equal to the supremum of the difference between their cumulative functions [15]. This distance takes values in the interval [0, 1]. It converges to zero if both distributions coincide. In all cases, the distance is smaller than 0.07. Thus, the distributions obtained by means of the "brute force" sampling and our method are quite similar for every single parameter.

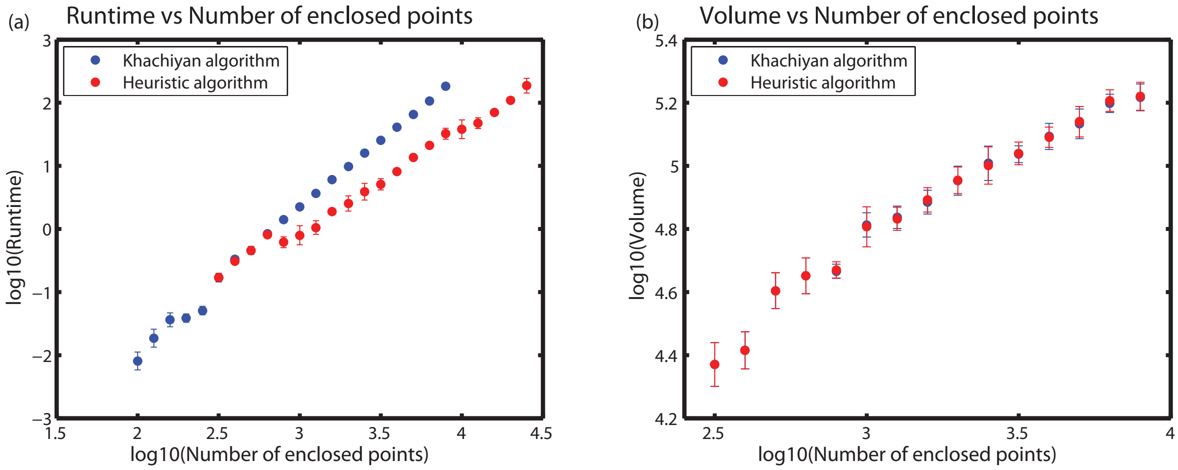

Figure 2. Comparison between the Khachiyan algorithm and our heuristic algorithm to determine minimum volume enclosing ellipsoids (MVEE). The left panel shows the runtime of both algorithms as a function of the number of points in a data set, for a space of 15 dimensions. The right panel displays the volume of the MVEE calculated as a function of the number of points in the data set in a space of 15 dimensions. In both figures, blue and red circles correspond to data derived from the Khachiyan algorithm and from our heuristic algorithm, respectively. In all cases shown, the test data set was composed of 100 groups of data points located far from each other, with equal numbers of random points distributed around the centre of each group. Note the superior efficiency of the heuristic algorithm (left panel), and its accurate volume estimation (right panel).

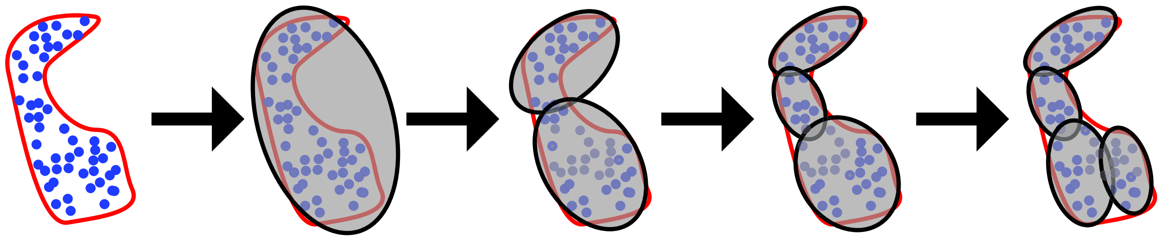

Figure 3. Clustering of data points for integration domain definition (hypothetical example). Viable volumes are generally nonconvex. Therefore, the single ellipsoid that encloses all the viable parameter points (second to left panel) will generally be much larger than the viable volume. It will typically also contain a high proportion of nonviable parameter points. Both features are undesirable. In the hypothetical example shown, when we group the viable points into 2 clusters, the sum of the ellipsoid volumes defined by them is smaller than in the case of a single cluster, but the two ellipsoids still enclose nonconvex regions, and are filled by a high proportion of nonviable parameters. In this hypothetical example, the viable space is well approximated by a set of three ellipsoids; therefore, after grouping the viable points into 3 clusters, the ellipsoids defined by them enclose mainly viable parameter points and the sum of their volumes is much smaller than in the case of two clusters but not much higher than the volume defined by 4 clusters.

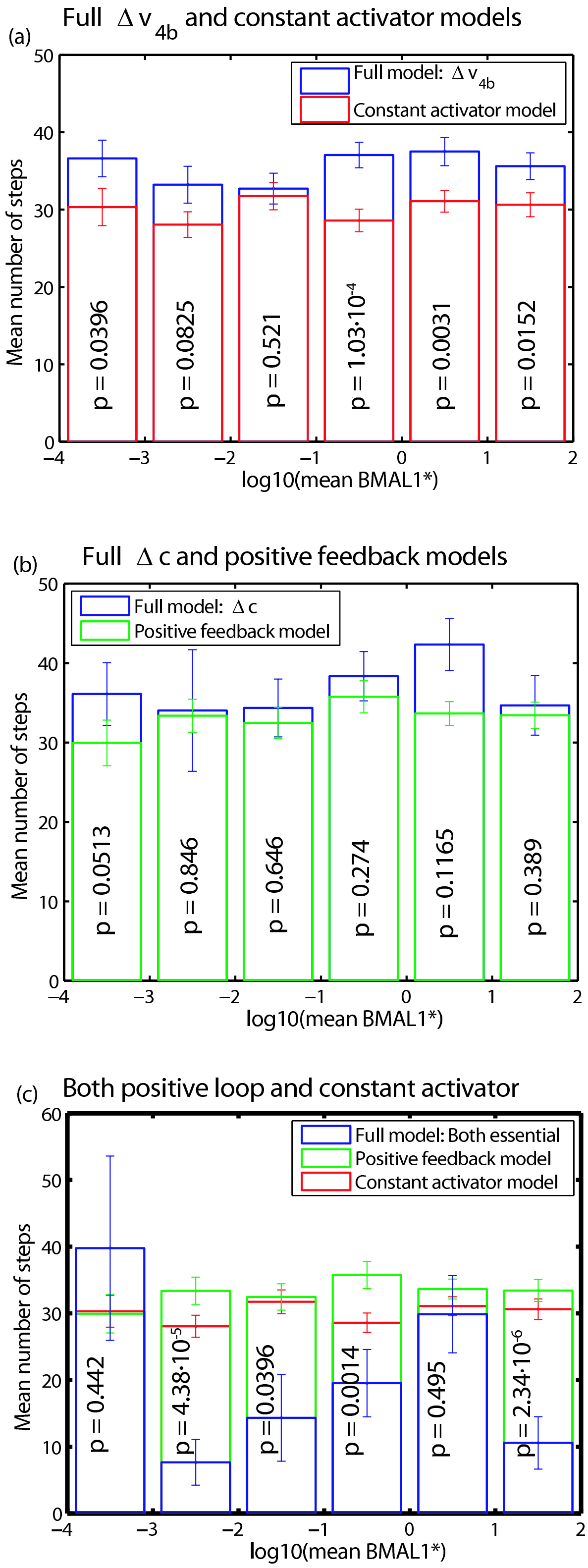

Figure 4. Local robustness: distribution of the mean number of random walk steps as a function of the mean BMAL1* concentration for different model architectures. Panel (a) shows the distributions of the mean number of steps for rhythmic Δv_{4b} viable parameter points of the full model (blue lines) and viable parameter points of the model with just a constant activator (red lines). Panel (b) shows the distributions of the mean number of steps for rhythmic Δc viable parameter points of the full model (blue lines) and viable parameter points of the model with just a positive feedback loop (green lines). Panel (c) shows the distributions of the mean number of steps for viable parameter points of the full model for which neither the constant activator and positive feedback loop can be removed (blue lines) and viable parameter points of the model with just a positive feedback loop and just a constant activator (green and red lines, respectively). We used the Wilcoxon rank sum test to calculate all the p-values shown in the figure.
